# CD11c-expressing Ly6C^+^CCR2^+^ monocytes constitute a reservoir for efficient *Leishmania* proliferation and cell-to-cell transmission

**DOI:** 10.1371/journal.ppat.1007374

**Published:** 2018-10-22

**Authors:** Sandrina Heyde, Lars Philipsen, Pauline Formaglio, Yan Fu, Iris Baars, Guido Höbbel, Corinna L. Kleinholz, Elena A. Seiß, Juliane Stettin, Patricia Gintschel, Anne Dudeck, Philippe Bousso, Burkhart Schraven, Andreas J. Müller

**Affiliations:** 1 Institute of Molecular and Clinical Immunology, Health Campus Immunology Infectiology and Inflammation (GC-I^3^), Otto-von-Guericke-University, Magdeburg, Germany; 2 Dynamics of Immune Responses Unit, Department of Immunology, Institut Pasteur, Paris, France; 3 Department of Immune Control, Helmholtz Centre for Infection Research, Inhoffenstrasse 7, Braunschweig, Germany; 4 Research Group Intravital Microscopy of Infection and Immunity, Helmholtz Centre for Infection Research, Inhoffenstrasse 7, Braunschweig, Germany; University of Calgary Cumming School of Medicine, CANADA

## Abstract

The virulence of intracellular pathogens such as *Leishmania major* (*L*. *major*) relies largely on their ability to undergo cycles of replication within phagocytes, release, and uptake into new host cells. While all these steps are critical for successful establishment of infection, neither the cellular niche of efficient proliferation, nor the spread to new host cells have been characterized *in vivo*. Here, using a biosensor for measuring pathogen proliferation in the living tissue, we found that monocyte-derived Ly6C^+^CCR2^+^ phagocytes expressing CD11c constituted the main cell type harboring rapidly proliferating *L*. *major* in the ongoing infection. Synchronization of host cell recruitment and intravital 2-photon imaging showed that these high proliferating parasites preferentially underwent cell-to-cell spread. However, newly recruited host cells were infected irrespectively of their cell type or maturation state. We propose that among these cells, CD11c-expressing monocytes are most permissive for pathogen proliferation, and thus mainly fuel the cycle of intracellular proliferation and cell-to-cell transfer during the acute infection. Thus, besides the well-described function for priming and activating T cell effector functions against *L*. *major*, CD11c-expressing monocyte-derived cells provide a reservoir for rapidly proliferating parasites that disseminate at the site of infection.

## Author summary

Infection with *Leishmania* parasites can result in chronic disease of several months duration, often accompanied with disfiguring and disabling pathologies. Central to *Leishmania* virulence is the capability to survive and multiply within professional phagocytes. While it is assumed that the parasites at some point have to exit the infected cell and infect new cells, the cycle of intracellular multiplication, release, and uptake into new host cells has never been studied in the ongoing infection. Therefore, it is unclear whether efficient growth of the pathogen takes place in a specific host cell type, or in a specific phase during the residency within, or during transfer to new cells. Here, we used a pathogen-encoded biosensor for measuring *Leishmania* proliferation in the ongoing infection, and in combination with a detailed analysis of the infected host cells involved. We could show that a monocyte-derived dendritic cell-like phagocyte subset, which is known for its role in inducing adaptive immune responses against *Leishmania*, represents a reservoir for efficient intracellular multiplication and spread to new host cells. These findings are important for our understanding of how the residency within a specific the cellular niche enables *Leishmania* parasites to efficiently multiply and persist at the site of infection.

## Introduction

Many pathogens have developed strategies to hijack host phagocytes and withstand their intracellular defense mechanisms. The ability to undergo cycles of replication within these phagocytes, release, and uptake into new host cells is central to the intracellular lifestyle, but has hardly been studied in the ongoing infection [1]. The parasite *Leishmania major* (*L*. *major*) represents such a well-adapted pathogen which can survive and replicate under the harsh microenvironmental conditions of endocytic compartments of professional phagocytes [2–4]. Although adaptive T cell responses increase the capability of phagocytes to control pathogens and limit further infection [5–7], *L*. *major* can prevail at the site of infection for extended periods of time. This can result in chronic infections of several months duration, often accompanied with disfiguring and disabling pathologies [8].

A complex host cell tropism is critical for efficient establishment of *L*. *major* infection. Flagellated promastigote forms of the parasite are rapidly taken up by neutrophils after inoculation of the skin [9]. Few days after infection, the parasites persist mainly within monocytes, macrophages and dendritic cells in the form of short, aflagellated amastigotes [9–12]. Activation of these phagocytes seems to differentially contribute to the control of *L*. *major* [11–17], and dampening of microbial proliferation is a crucial containment mechanism of the immune system for controlling the intracellular pathogens [18,19]. Specifically, nitric oxide produced by the inducible nitric oxide synthase iNOS can non-lethally slow down *L*. *major* replication rates [18], a mechanism that has been shown to be enhanced during secondary infections [12].

While activated dendritic cell-like populations have been shown to harbor a large fraction of the parasite in the infected skin [10,11], more recent studies focused on the characterization of monocyte subsets, showing an important role of inflammatory monocytes as a niche for the parasite during primary infection, and for efficient containment of *L*. *major* during secondary infections [12]. Moreover, monocytes cells have been shown to promote parasite survival and suppress clearance of *Leishmanial donovani* during visceral leishmaniasis [20,21].

Despite these findings, it has remained very difficult to dissect the different host cell types infected by *L*. *major* in the infected skin regarding their permissiveness for rapid parasite proliferation in vivo. This is of particular interest since recent studies suggest that different pools of high and low proliferating *L*. *major* coexist at the site of infection [18,22]. However, a side-by-side characterization of the phenotypes of the cell types harboring parasites of rapid versus slow proliferation rates has been lacking so far. Furthermore, pathogen burden increases a thousand fold between inoculation and the peak of the infection, and can remain high over weeks even after the onset of a protective gamma interferon (IFN-γ)-dominated T cell response [23]. Thus, pathogen proliferation must fuel the spread of the amastigotes to new host cells at the site of infection. However, while the uptake mechanisms for both promastigotes and amastigotes into host phagocytes have been intensely studied in vitro [24–26], knowledge on the dynamics of the spread at the site of infection is scarce. Also, any link between distinct pathogen proliferation rates and in vivo cell-to-cell transmission has remained unexplored. Yet, this would be crucial to understand mechanisms which could drive, or impede, the cycle of *L*. *major* replication, release, and uptake into new host cells.

Here, we employed a genetically encoded proliferation biosensor to investigate the cellular niches of high versus low *L*. *major* proliferation in the ongoing cutaneous infection, and to characterize pathogen proliferation rates in the context of transmission to new host cells. We found that parasite proliferation rates were homogenous within individual host cells, however varied substantially between different infected phagocytes. Specifically, monocyte-derived dendritic cell-like, Ly6C^+^CCR2^+^ monocytes with high CD11c expression were harboring parasites with the highest proliferation rates at the site of infection, and were highly overrepresented among infected cells in the acute infection. In contrast, newly recruited phagocytes were, irrespective of their cell type, preferentially infected by high proliferating parasites. This suggests that CD11c-expressing monocytes infected by high proliferating *L*. *major* serve as a reservoir for pathogen spread to all new host phagocytes, while other phagocyte populations are less efficient in fueling *L*. *major* replication and dissemination at the site of infection.

By revealing specific niches and host cell tropisms for high proliferating *L*. *major*, our findings delineate the impact of a specific physiological parameter of a pathogen on its interaction with the host immune system. This provides a critical contribution to our understanding of how intracellular pathogens establish infection and counteract the containment by the immune response.

## Results

### *L*. *major* proliferation is heterogenic on a tissue level but cell intrinsically homogenous

**Fig 1:**
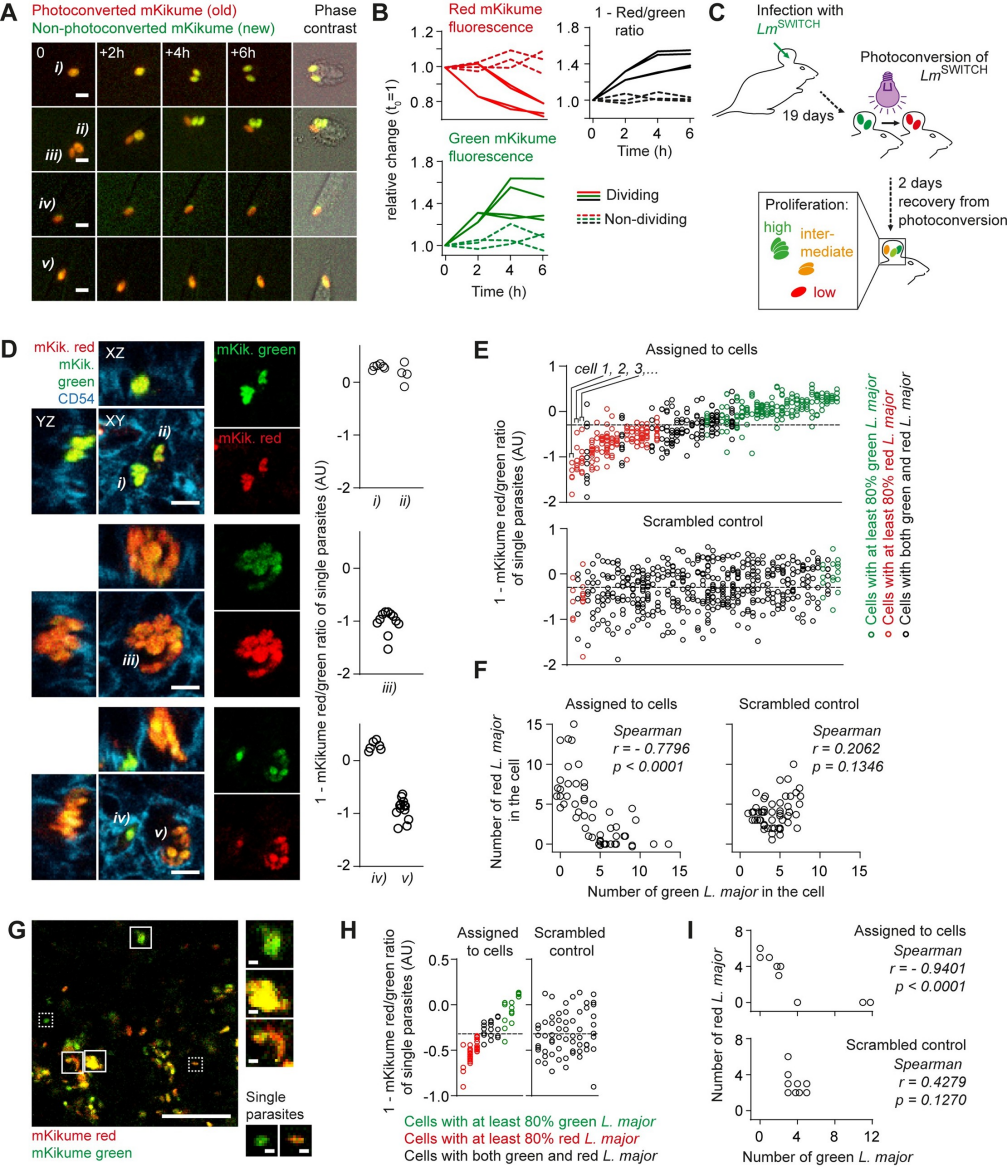
Photoconversion-based proliferation measurements show cell-intrinsic control of *L*. *major* proliferation rates in vivo. **(A)** Peritoneal macrophages infected with *Lm*^SWITCH^ in vitro, photoconverted and imaged over time. i, ii, parasites undergoing cell division in the course of the movie, iii, iv, v, non-dividing parasites. Scale bar, 5 μm. **(B)** Quantification of relative changes of mKikume red and green fluorescence, and 1 –red/green ratio from parasites i) through v) from (A). Each line represents one parasite; solid lines, parasites which undergo division in the course of the movie; dashed line, non-dividing parasites. **(C)** Experimental setup for in vivo *L*. *major* proliferation measurement using *Lm*^SWITCH^. **(D)** Immunofluorescence analysis of *Lm*^SWITCH^ infected C57BL/6 mouse ears photoconverted as shown in (C). Tissues were stained anti-CD54 to mark cell outlines, and red/green ratios of the individual parasites within the cells (i-v) were determined (graphs, each symbol represents one parasite segmented manually from at least 3 different Z slices of the stack shown on the left). Scale bar, 5 μm. Values represented as 1 –red/green fluorescence ratio. **(E)** Quantification of single parasite red/green ratios (one symbol per parasite) infecting individual cells (one cell is represented in each column), ordered according to the mean red/green fluorescence ratio of parasites in the respective column (upper panel and scrambled control with individual parasites assigned randomly to the cells (lower panel). Red symbols, >80% parasites below the overall mean, green symbols, >80% parasites above the overall mean. The overall mean is represented by a dotted line. Values represented as 1 –red/green fluorescence ratio. **(F)** Left panel, number of *Lm*^SWITCH^ which are mainly red within a single cell plotted against the number of mainly green *Lm*^SWITCH^ within the same cell. Right panel, scrambled control as described in (E). Each symbol represents one infected cell analysed. **(G)** Intravital 2-photon analysis of *Lm*^SWITCH^ infected C57BL/6 mouse ears that were photoconverted as shown in (C). Solid boxes, groups of more than 5 parasite-sized signals localized closely enough to be in the same host cell, dotted boxes, individual (less than 3) parasites. Scale bars, 50 μm (large image), 3 μm (insets). **(H-I)** Analysis of mKikume red/green ratio of *Lm*^SWITCH^ within individual parasite clusters as described in (E-F). Examples representative of >5 animals imaged.

Many pathogens, including *Leishmania* spp., do not proliferate uniformly at the same rate during an infection, but occur as heterogenic populations of high and low proliferating microbes [22,27–29]. However, analyzing pathogen proliferation on a cellular level, i.e. in combination with immunofluorescence, had remained challenging [22,30]. To overcome this limitation, we set out to characterize *L*. *major* proliferation using a photoconversion-based in vivo proliferation biosensor [18,31]. The system relies on the photoconvertible mKikume protein constitutively expressed in *L*. *major* (*Lm*^SWITCH^). In its native form, mKikume exhibits green fluorescence (excitation/emission: 488nm/515nm), but can be photoconverted to red fluorescence (excitation/emission: 561nm/590nm) using a light pulse of 405 nm[32]. The dilution rate of the red, photoconverted protein strictly and quantitatively correlates with cell division, whereas high proliferation is associated with more production of green, non-photoconverted protein [18]. Consequently, the ratio between diluted, photoconverted red fluorescence and newly expressed green fluorescence can be used to measure *Lm*^SWITCH^ proliferation 48h after a photoconversion applied to the parasite (**Fig 1A and 1B**). We employed this system to determine *L*. *major* proliferation rates in vivo three weeks post infection of the ear dermis (**Fig 1C**)[18]. For this, we infected C57BL/6 mice intradermally in the ear with *Lm*^SWITCH^. After three weeks, the sites of infection were photoconverted, and ears were harvested 48h after photoconversion, fixed and embedded for cryosectioning. Using confocal immunofluorescence microscopy, we observed that *L*. *major* proliferation rates were very similar among the parasites within individual infected cells. However, the pathogen proliferation rates between different host cells varied dramatically (**Fig 1D**). In order to quantify this cell-specifically distinct proliferation, we determined in 50 infected cells the red and green mKikume fluorescence of each individual parasite. We could observe that the infected cells predominantly contained parasites either above or below the mean red and green fluorescence ratio over all parasites analyzed (**Fig 1E**, upper graph). Of note, this was not the case in a scrambled analysis where the parasites were arbitrarily assigned to the cells (**Fig 1E**, lower graph). In line, we observed a strong negative correlation between the number of red and green parasites within individual cells (**Fig 1F**). Intravital 2-photon imaging of mouse ear tissue infected and photoconverted using the same conditions also clearly revealed clusters of high proliferating parasites separated from clusters of low proliferating parasites (**Fig 1G–1I**). Thus, we concluded that distinct pathogen proliferation rates are linked to specific cellular niches.

### CD11c defines a phagocyte subset that harbors highly proliferative *L*. *major*

**Fig 2:**
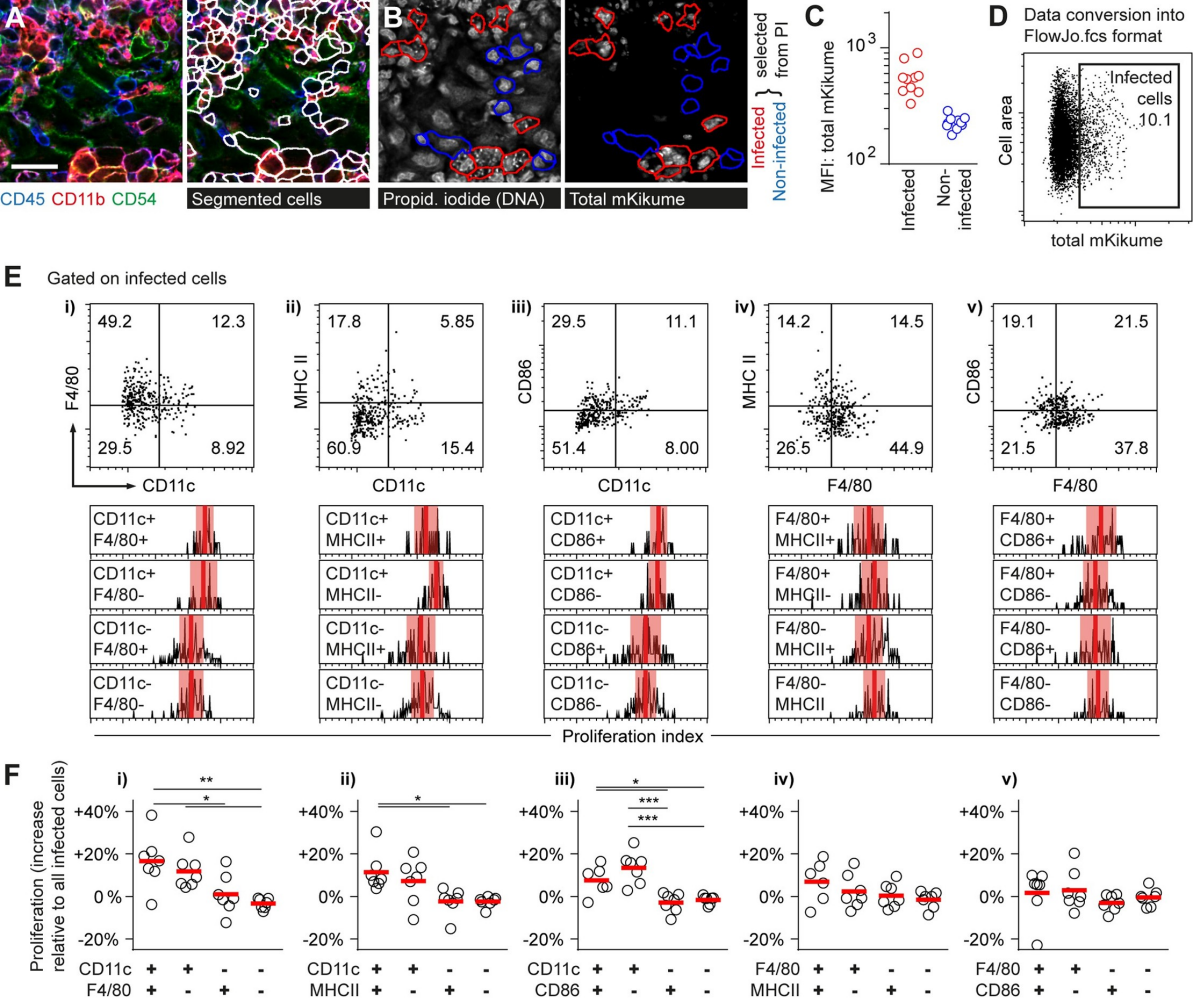
CD11c defines a phagocyte subset harboring highly proliferative *L*. *major*. **(A-F)** *Lm*^SWITCH^-infected ears were photoconverted 3 weeks post infection and analyzed 48h later via MELC [34]. **(A)** Automated cell segmentation using the RACE software [35] based on CD45, CD54 and CD11b signals. Scale bar, 20 μm. **(B-C)** Examples (B) and quantification (C) of infected cells (red outlines and symbols) and non-infected cells (blue outlines and symbols) identified manually by propidium iodide staining of the small parasite nuclei. **(D)** Conversion to FlowJo.fcs format allows for gating on infected cells according to the threshold defined in (C), see also S1 Fig **(E)** Top row: Infected cells subdivided according to CD11c versus F4/80, MHC II or CD86 expression (i, ii, iii) and F4/80 versus MHC II and CD86 (iv, v). Bottom row: Relative proliferation rates of *Lm*^SWITCH^ (obtained by normalization of the subpopulation’s proliferation indices to the overall mean proliferation index within each sample) in the respective subpopulations. Vertical bars denote the mean; shaded red boxes, standard deviation. **(F)** Quantification of 7 individual MELC experiments performed in 4 different infected mice according to the populations defined in (E), i though v. Each symbol represents one imaged infection site. Horizontal bars represent the mean. ***p < 0.001; **p < 0.01; *p < 0.05. Vertical bars denote the mean.

Recruited monocytes, monocyte-derived macrophages and dendritic cells, as well as neutrophils have been shown to constitute the main infected cell types in an ongoing *L*. *major* infection [10–12,33]. However, it had been impossible to show in vivo whether any of these cell types preferentially harbors parasites of a distinct proliferation rate, which would be critical to dissect niches permissive for pathogen proliferation from phagocytes that restrict the growth and dissemination of *L*. *major* at the site of infection [22]. In order to characterize *L*. *major* infected cell types *in situ*, we analyzed ear tissue sections infected for three weeks with *Lm*^SWITCH^ by Multi-Epitope Ligand Cartography (MELC), a multiparameter microscopy approach based on consecutive immunofluorescence staining/bleaching cycles [34]. In brief, cryosections fixed and embedded 48h after photoconversion were imaged to detect both red and green mKikume in *L*. *major*, photobleached, and, in subsequent automated immunofluorescence staining/photobleaching cycles, probed for expression of a series of surface markers at the same tissue site. With each cycle, a transmitted light image was recorded to which the fluorescence images were aligned. Antibodies against CD45, CD54, CD11b, CD11c, F4/80, CD86, MHCII, CD45R, as well as propidium iodide were found to be compatible with the fixation conditions needed for the preservation of the mKikume protein. (**S1 Fig** Panel A). Cell outlines were automatically defined from the CD45, CD11b and CD54 images, and mean cell body and outline fluorescence values were normalized and converted into cytometry data [35,36] (**Fig 2A, S1 Fig** Panels B-C). To detect cells infected by *L*. *major*, combined red and green mKikume fluorescence (total mKikume) in infected and non-infected cells manually selected from the propidium iodide DNA staining were analyzed (**Fig 2B and 2C**). This allowed for determination of a total mKikume threshold applicable to images acquired in different experiments and different tissue depths (**Fig 2D, S1 Fig** Panel D). Likewise, manually selected cells from three different experiments served to define a threshold for CD11c, F4/80, CD86, MHCII as markers for characterization of infected phagocytes [11], and CD45R as a marker unrelated to the different monocyte subsets (**S1 Fig** Panel E). Thus, we could reliably and automatically determine the localization of *Lm*^SWITCH^ in the infected tissue in conjunction with multiplex analysis of the parasite’s host cell.

We then gated for each marker on a positive and a negative population of infected cells, and compared *L*. *major* proliferation rates depending on each individual marker (**S1 Fig** Panel F). We observed that infected cells positive for CD11c were enriched in high proliferating pathogens, whereas all other markers tested were not per se specific for a distinct pathogen proliferation rate (**S1 Fig** Panel G). In order to find differences in pathogen proliferation within subsets of CD11c-positive and negative phagocyte subsets, we performed combined marker analysis, in which *L*. *major* proliferation was assessed in subpopulations of cells differentially expressing CD11c or F4/80 in combination with class II MHC or CD86 (**Fig 2E**). Importantly, also when analyzed in combination with CD11c expression, F4/80, class II MHC and CD86 expression were not characteristic of a distinct pathogen proliferation rate (**Fig 2E and 2F**, i-iii). Likewise, analysis of F4/80-positive and negative subpopulations regarding class II MHC and CD86 expression revealed no significant change of pathogen proliferation in any subset analyzed (**Fig 2E and 2F**, iv, v).

**Fig 3:**
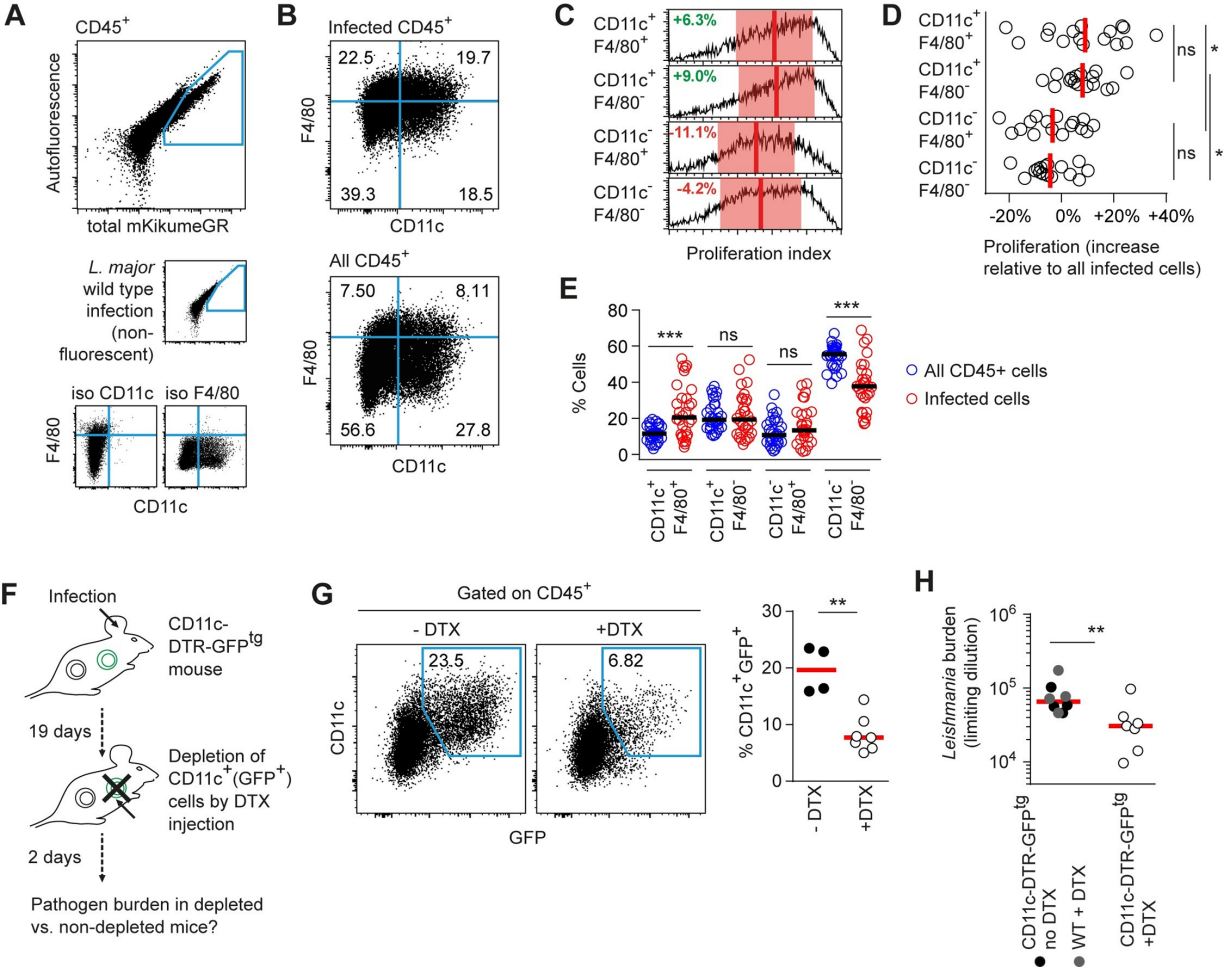
CD11c^+^ cells represent a niche for maintaining a high *L*. *major* burden. **(A-B)** C57BL/6 mice ears were infected intradermally with *Lm*^SWITCH^. 3 weeks post infection, parasites in the mouse ear were photoconverted and 48h later ears were harvested for flow cytometry analysis of intracellular *L*. *major* proliferation. **(A)** Strategy to identify *L*. *major*-infected cells (large plot) and CD11c^+^ and F4/80^+^ cell subsets **(B)** using controls infected with non-fluorescent *L*. *major* wildtype parasites (A, middle row, small plot) and isotype controls (A, lower row, small plots). **(C)** Comparison of proliferation rates of *Lm*^SWITCH^ located in the different cell compartments defined by F4/80 and CD11c. Vertical bars denote the mean, and shaded red boxes the standard deviation. **(D)** Quantitative analysis of the proliferation rates of *Lm*^SWITCH^ within the different cell populations shown in B-C. Each dot represents one mouse ear. Relative proliferation indices were obtained by normalization of the subpopulation’s proliferation index to the overall mean proliferation index within each sample. Vertical bars represent the mean. *p < 0.05; ns, not significant. Data pooled from three independent experiments. **(E)** Distribution of CD11c and F4/80 expression within infected (red symbols) and non-infected (blue symbols) CD45^+^ cells. Data shown are combined from the experiments shown in (D) and S2 Fig. Horizontal bars represent the mean. ***p < 0.001; ns, not significant. **(F)** Experimental approach for temporary Diphtheria toxin (DTX)-mediated CD11c^+^ cell depletion using the CD11c-DTR-GFP^tg^ mouse model. **(G)** Quantification of the efficiency of depletion by measuring the percentages of GFP^+^CD11c^+^ cells in CD45^+^ cells isolated from infected mouse ears treated as shown in (F). **(H)** Limiting dilution analysis of *L*. *major* tissue burden in the ears of 3 week-infected CD11c-DTR-GFP^tg^ mice, either PBS-injected (closed black symbols) or injected with DTX (open circles) 48h prior to analysis, or nontransgenic animals injected with DTX (closed grey symbols). **p<0.01. Each dot represents one individual mouse ear.

In order to analyze the cellular niche of proliferating *L*. *major* via flow cytometry, we infected C57BL/6 mice intradermally in the ear with *Lm*^SWITCH^. After three weeks, the sites of infection were photoconverted, and ears were harvested 48h after photoconversion for tissue homogenization and flow cytometry analysis of *Lm*^SWITCH^ proliferation in infected cells. To enable an undisturbed ratiometric analysis of mKikume recovery after photoconversion by flow cytometry, we chose a limited set fluorophores with both excitation (ex) and emission (em) spectra different from the two forms of mKikume (green, ex/em 488nm/515nm and red, ex/em 561nm/590nm). This strategy limited the number of antibodies for staining to two to three, but efficiently excluded spectral overlap with the reporter system. The isolated cells were thus stained anti-CD45 to identify leukocytes, as well as anti-CD11c and anti-F4/80 to analyze subpopulations comparable to MELC analysis **(Fig 3A and 3B)**. In perfect accordance with the MELC data, *L*. *major* proliferation was significantly higher in CD11c^+^F4/80^+^ and CD11c^+^F4/80^-^ cells compared to CD11c-negative cell populations, whereas F4/80 expression did not correlate with pathogen proliferation (**Fig 3C and 3D**). To exclude any residual spectral overlap of the antibody staining or cell-type-specific autofluorescence as an explanation for the different proliferation rates observed in the distinct cell populations, a scrambled control with arbitrary photoconversion right before proliferation measurement was analyzed. To this end, *Lm*^SWITCH^ in the ear were photoconverted at random by using a grid (**S2 Fig** Panel A). In these controls, host cell CD11c expression and pathogen proliferation index were no longer correlated (**S2 Fig** Panels B-C). Thus, a possible spectral overlap between marker staining and mKikume red and green fluorescence readout was not responsible for the enrichment in parasites exhibiting the high proliferation observed in CD11c^+^ cells. When we compared the distribution of CD11c and F4/80-expression among infected and non-infected host cells, we observed that the CD11c^+^F4/80^+^ population was significantly overrepresented among the infected cells (**Fig 3E**). Thus, in the acute infection, *L*. *major* exhibits high proliferation rates preferentially in CD11c^+^ cells, and is overrepresented in CD11c^+^F4/80^+^ phagocytes.

In order to test the effect of a short-term absence of CD11c^+^ cells on pathogen burden, we employed the CD11c-DTR-GFP^tg^ model [37], in which cell-specific expression of the human Diphtheria toxin receptor (DTR) mediates susceptibility to Diphtheria toxin (DTX) in CD11c^+^ cells (**Fig 3F**). This enabled us to specifically deplete CD11c-expressing cells, which are also identifiable by GFP-expression in this mouse model, for 48h in an on-going infection (**Fig 3G**). Strikingly, the depletion of the CD11c^+^ cells resulted in a significant reduction in pathogen burden, underlining that the presence of these cells might represent a niche in which *L*. *major* can efficiently proliferate in order to maintain parasite numbers at the site of infection (**Fig 3H**). Taken together, these results suggest that CD11c^+^ cells represent a niche in which *L*. *major* can efficiently proliferate.

### CD11c-expressing Ly6C^+^CCR2^+^ monocytes are the main cellular niche of high *L*. *major* proliferation

**Fig 4:**
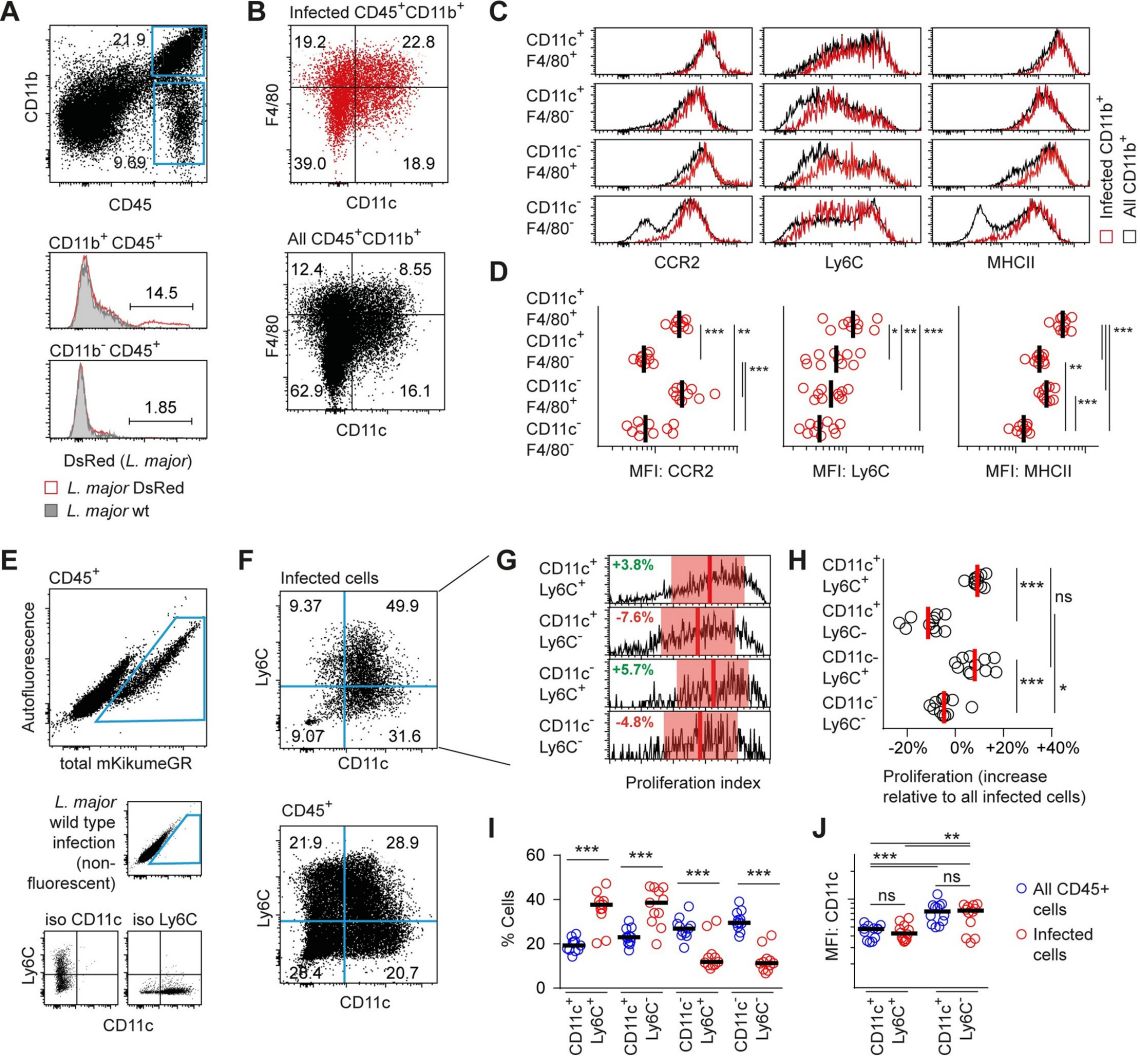
CD11c-expressing Ly6C^+^CCR2^+^ monocytes are the main cellular niche of high *L*. *major* proliferation. **(A-D)** C57BL/6 mice were infected for 3 weeks with monofluorescent DsRed-expressing *L*. *major* compatible with multicolor flow cytometry. **(A)** Among CD45^+^ cells, mainly the CD11b^+^ cells were found to be infected with *L*. *major*. **(B-C)** Cell populations defined by CD11c and F4/80 expression were further analyzed with respect to expression of CCR2, Ly6C, and MHC class II. Red plots and histograms, infected cells; black plots and histograms, all cells. **(D)** Quantitative analysis of marker expression shown in (C) for infected cells. Vertical bars represent the mean. *p < 0.05, **p < 0.01, ***p < 0.001; each symbol represents one infected ear per plot. **(E-J)** C57BL/6 mice ears were infected intradermally with *Lm*^SWITCH^. 3 weeks post infection, parasites in the mouse ear were photoconverted and 48h later ears were harvested for flow cytometry analysis of intracellular *L*. *major* proliferation. **(E)** Strategy to identify *L*. *major*-infected cells (large plot) and **(F)** Ly6C^+^ and CD11c^+^ cell subsets using controls infected with non-fluorescent *L*. *major* wildtype parasites (E, middle row, small plot) and isotype controls (E, lower row, small plots). **(G)** Comparison of proliferation rates of *Lm*^SWITCH^ located in the different cell compartments defined by Ly6C and CD11c. Vertical bars denote the mean, and shaded red boxes the standard deviation. **(H)** Quantitative analysis of the proliferation rates of *Lm*^SWITCH^ within the different cell populations shown in F-G. Each dot represents one mouse ear. Relative proliferation indices were obtained by normalization of the subpopulation’s proliferation index to the overall mean proliferation index within each sample. Vertical bars represent the mean. *p < 0.05, ***p < 0.001; ns, not significant. **(I)** Distribution of Ly6C and CD11c expression within infected (red symbols) and non-infected (blue symbols) CD45^+^ cells. Horizontal bars represent the mean. ***p < 0.001. **(J)** Absolute mean fluorescence intensity of the CD11c^+^Ly6C^+^ and CD11c^+^Ly6C^-^ populations as defined in (F). **p < 0.01, ***p < 0.001; ns, not significant. Each symbol represents one mouse ear per plot. Data pooled from three independent experiments.

The different subsets of phagocytes derived from monocytes after recruited to the skin exhibit a high degree of functional specialization and have been assigned divergent roles in the control of *L*. *major* infection[11,12,15,38]. In order to better characterize the infected subpopulations, we sought to analyze the populations defined in MELC by CD11c and F4/80 using the monocyte markers Ly6C, CCR2 and CD11b, as well as MHC class II. For this, we used a monofluorescent DsRed-expressing *L*. *major* compatible with multicolor flow cytometry [39]. As expected, CD11b-positive cells were the main infected cell population [10–12], which were subdivided according to CD11c and F4/80 expression for the analysis of the remaining markers (**Fig 4A and 4B**). Strikingly, the CD11c^+^F4/80^+^ population exhibited the highest expression of Ly6C and CCR2, suggesting these cells were mainly inflammatory monocytes [12](**Fig 4C and 4D**). Interestingly, this population also exhibited high levels of MHC class II, indicating that the CD11c^+^F4/80^+^ phagocytes might include monocyte-derived dendritic cells [11,17]. In contrast, the CD11c^+^F4/80^-^ and CD11c^-^F4/80^+^ populations exhibited intermediate Ly6C and low CCR2, or Low Ly6C and high CCR2 expression, respectively, indicating that these included recently recruited inflammatory monocytes, and, for the CD11c^-^F4/80^+^, monocyte-derived macrophages [11,12,38].

Interestingly, although their mean Ly6C expression level was lower, we found that a part of the cells in the CD11c^+^F4/80^-^, the CD11c^-^F4/80^+^, and the double-negative population expressed high levels of Ly6C. This prompted us to analyze *L*. *major* proliferation in the context of Ly6C and CD11c (**Fig 4E and 4F**). As expected, we found a very high proliferation in the infected CD11c^+^Ly6C^+^ population, but interestingly, also within CD11c^-^Ly6C^+^ cells, indicating that among the monocyte-derived phagocytes, not only the CD11c^+^ subpopulation exhibited high *L*. *major* proliferation (**Fig 4G and 4H**). However, when we compared the distribution of CD11c versus Ly6C of infected versus non-infected cells, we observed that CD11c^+^Ly6C^+^ cells were substantially overrepresented, whereas CD11c^-^Ly6C^+^ cells, despite the high *L*. *major* proliferation within them, were severely underrepresented among infected phagocytes (**Fig 4I**). This suggests that although high proliferating *L*. *major* are observed also within other cell types, the CD11c^+^Ly6C^+^ cells represent the main niche for high proliferating pathogens. Also, as indicated by the higher absolute CD11c expression, the CD11c^+^Ly6C^-^ population is likely to include the dermal dendritic cells [38] (**Fig 4J**). Therefore, we concluded that monocyte-derived dendritic cell-like, CD11c-expressing Ly6C^+^CCR2^+^ phagocytes represent the main niche for high proliferating pathogens.

### Newly infected host cells predominantly harbor high proliferating *L*. *major*

**Fig 5:**
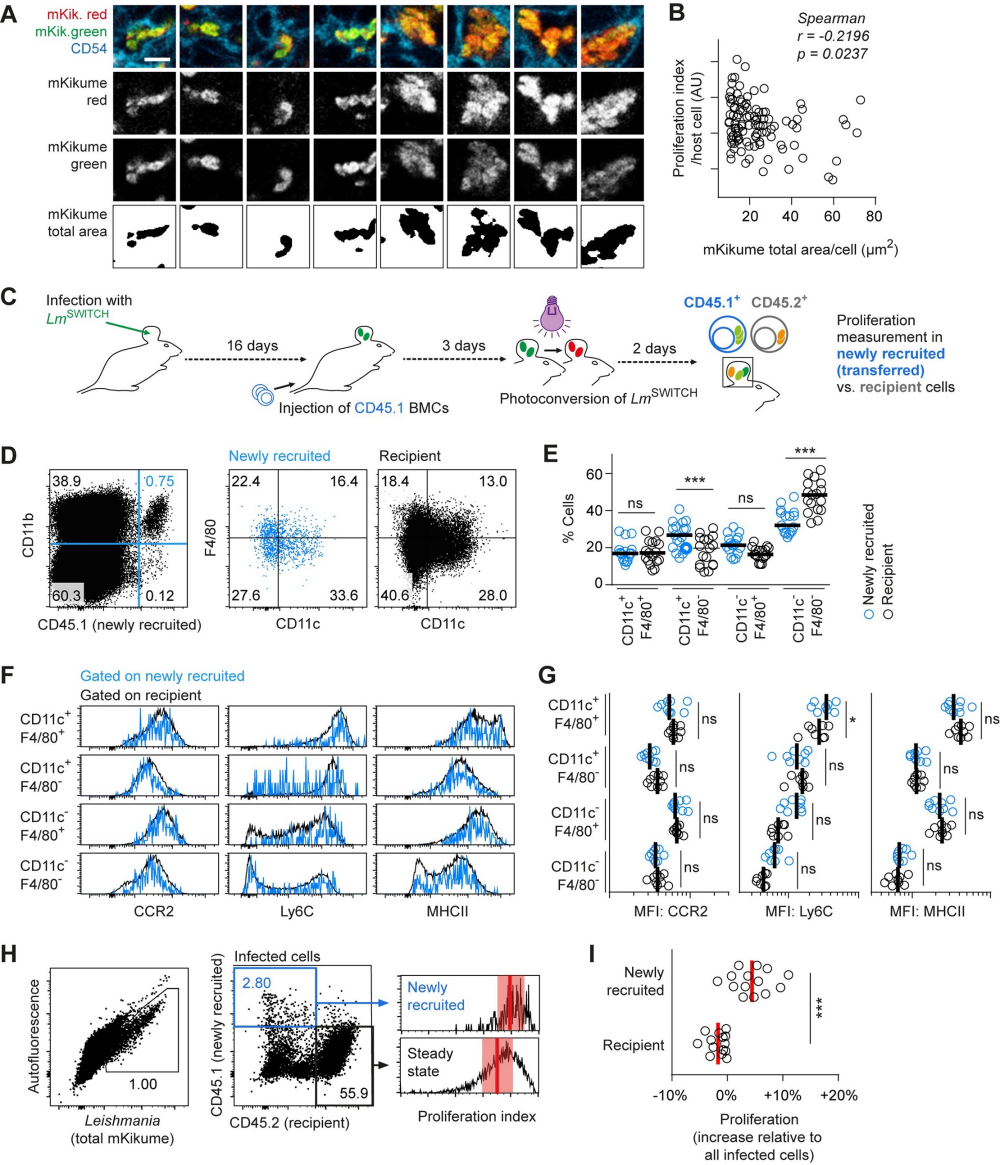
Newly infected host cells predominantly harbor high proliferating *L*. *major*. **(A-B)** *Lm*^SWITCH^ infected C57BL/6 mouse ears were photoconverted 3 weeks post infection and analyzed 48h later confocal immunofluorescence microscopy. Mean pathogen proliferation within individual cells was plotted against the total mKikume-positive area per confocal section through the cells. Raw data reanalyzed from Fig 1D–1F. Scale bar, 5 μm. **(C)** Experimental strategy to determine *L*. *major* proliferation in phagocytes newly recruited (CD45.1^+^) to the site of infection as compared to phagocytes of the host (CD45.2^+^). **(D-E)** Characterization of CD11c and F4/80 expression in CD11b^+^ cells of the newly recruited (blue symbols and graphs) versus recipient (black symbols and graphs) population. Horizontal bars represent the mean. **p < 0.01, ***p < 0.001; ns, not significant. Each symbol represents one mouse ear. Data pooled from the analyses of Ly6C, and CCR2 and MHC class II analysis experiments (see below). (**F)** Newly recruited versus recipient subpopulations defined by CD11c and F4/80 expression were further analyzed with respect to expression of CCR2, Ly6C, and MHC class II. Blue histograms, newly recruited CD45.1^+^ cells; black plots and histograms, recipient CD45.2^+^ cells. **(G)** Quantitative analysis of marker expression shown in (F) for infected cells. Vertical bars represent the mean. *p < 0.05; ns, not significant. Each symbol represents one infected ear per plot. Data in (F-G) show separate experiments for the analysis of CCR2 and MHC class II, or Ly6C, respectively. **(H-I)** *Lm*^SWITCH^ infected C57BL/6 (CD45.2^+^) mice were injected at 16 days p.I. with CD45.1^+^ mone marrow cells photoconverted at 18 days p.I. and analyzed 48h later by flow cytometry. **(H)** Gating strategy for detection of infected CD45.1^+^ newly recruited and CD45.2^+^ recipient infected cells isolated from the site of infection (left and middle panel). Proliferation rates of the infected newly recruited and recipient population of one example ear are shown as histograms (right panel). Vertical bars denote the mean, and shaded red boxes the standard deviation. **(I)** Quantitative analysis of *Lm*^SWITCH^ proliferation rates. Relative proliferation rates in newly recruited or recipient cells were obtained by normalization of the subpopulation’s proliferation indices to the overall mean proliferation index within each sample. Each dot represents one mouse ear. Vertical bars denote the mean. ***p < 0.001. Data pooled from three independent experiments.

Next, we analyzed whether cells infected with high proliferating *L*. *major* would, as a result, be populated with more parasites per cell. However surprisingly, we found a slight but significant negative correlation between *L*. *major* proliferation rates within individual cells and the respective cellular parasite burden determined from confocal microscopy. This suggested that high proliferating parasites were present at lower numbers per infected cell (**Fig 5A and 5B**). One explanation for this could be that high proliferating *L*. *major* are more efficiently released from infected cells, and thus might represent the main parasite population infecting new host cells. In order to test whether newly infected cells preferentially harbor high proliferating pathogens, we synchronized the arrival of newly recruited cells by adoptive bone marrow transfer [11,33]. For this, C57BL/6 mice (CD45.2^+^) infected for 16 days with *Lm*^SWITCH^ were injected with 10^8^ CD45.1^+^ bone marrow cells. The *Lm*^SWITCH^-infected ears were photoconverted 19 days post infection (p.I.), and analyzed at day 21 p.I.. Thus, pathogen proliferation in infected newly recruited cells (CD45.1^+^) could be compared to pathogen proliferation in CD45.2^+^ recipient cells, which, on average, are expected to be present at the site of infection earlier than the transferred CD45.1^+^. (**Fig 5C and 5D**). Of note, a shorter time frame for CD45.1^+^ cell recruitment did not yield enough infected cells for proliferation analysis (**S3 Fig** Panels A-B). Besides a higher content of CD11c^+^F4/80^-^ cells (**Fig 5E**) and slightly elevated Ly6C expression of the CD11c^+^F4/80^+^ population (**Fig 5F and 5G**), the newly recruited cells had very similar phenotype compared with the recipient cell population, suggesting the newly recruited cells had differentiated in all different monocyte-derived subsets present at the site of infection. However strikingly, we observed that pathogen proliferation in newly recruited CD45.1^+^ cells was significantly higher than in CD45.2^+^ recipient cells (**Fig 5H and 5I**). Switched color experiments were performed to exclude an influence of the antibody staining on proliferation measurements, and yielded the same results (**S3 Fig** Panels C-D). This suggests that high pathogen proliferation occurs in the context of the infection of new host cells.

### High proliferating *L*. *major* preferentially undergo cell-to-cell transmission

**Fig 6:**
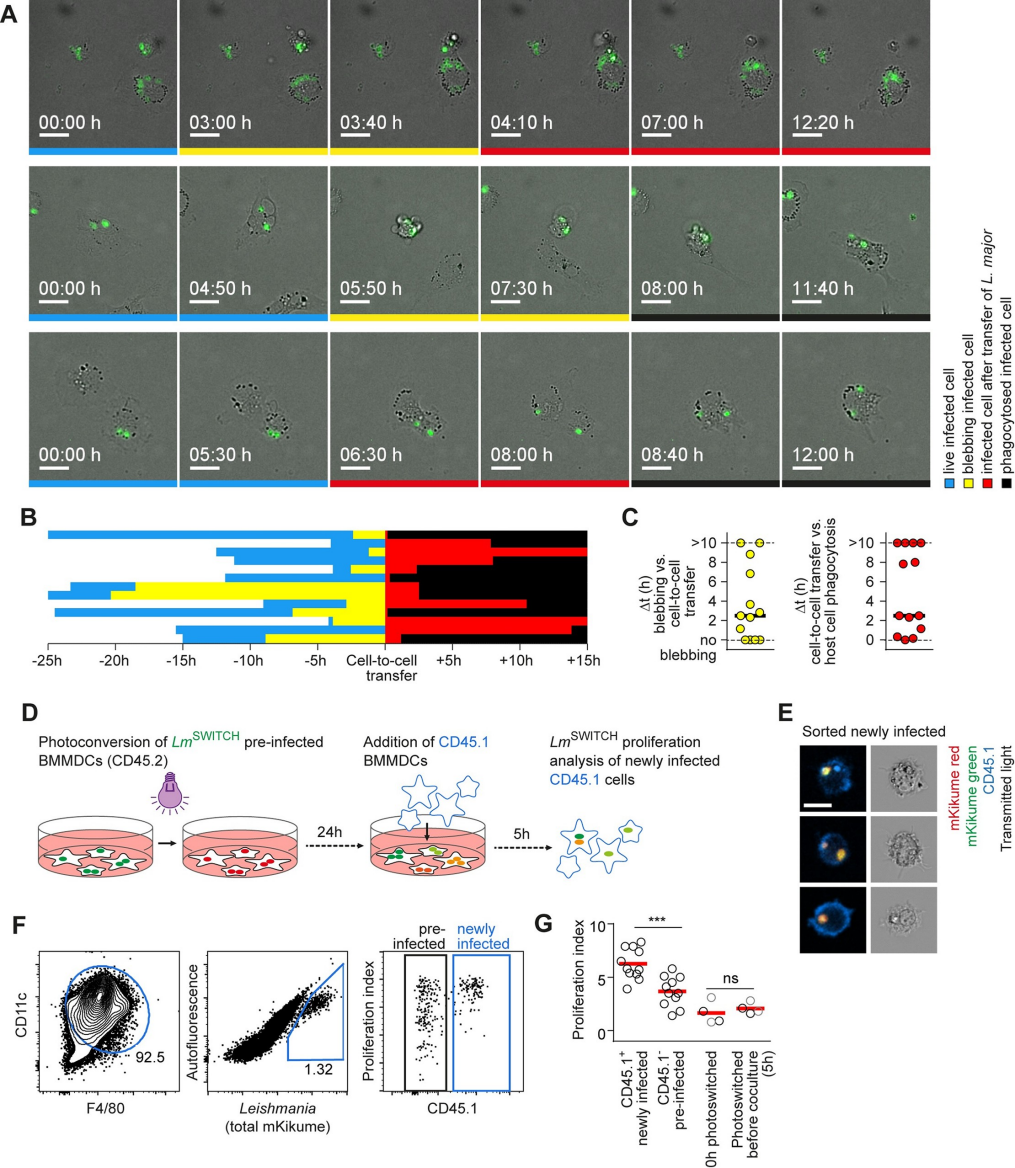
High proliferating *L*. *major* preferentially undergo cell-to-cell transmission. **(A-C)** Time lapse videomicroscopy of intraperitoneal macrophages infected for 24 h with fluorescently labeled *L*. *major*. **(A)** Three examples of cell-to-cell transfer between two phagocytes. Selected frames of a time series spaced 10 min are shown. Frames in which the originally infected cell is still alive and motile are marked blue, frames showing the original phagocyte with signs of cell death before transfer of the parasite are marked in yellow, after transfer in red. Complete phagocytosis of the original host cell is marked black. Scale bar, 10 μm. **(B)** Analysis of the sequence of host cell death, parasite transfer and phagocytosis of the original host cell from 13 cell-to-cell transfer events analysed, using the colour code indicated in (A). **(C)** Quantification of the time between the first blebbing as a sign of cell death and cell to cell transfer (left) and the time between cell-to-cell transfer and complete phagocytosis of the original host cell. Horizontal bars denote the median. **(D-G)** Quantitative in vitro analysis of parasite cell-to-cell transfer in mixed of bone marrow-derived macrophages and dendritic cells (BMMDCs). **(D)** Experimental setup for in vitro cell-to-cell transfer analysis. **(E)** Confocal microscopy of sorted CD45.1^+^ infected cells (newly infected) showing intracellular localization of fluorescent *L*. *major*. Images are representative of > 20 cells inspected microscopically. Scale bar, 10 μm. **(F)** Gating strategy to identify newly infected (CD45.1^+^) and preinfected (CD45.1^-^) BMMDCs. **(G)** Quantification of proliferation rates in newly infected (CD45.1^+^) and preinfected (CD45.1^-^) cells analysed as shown in (A) (left), and controls photoconverted 0h and 5h before analysis to assess the recovery from photoconversion occurring in the 5h of coculture (right) in newly infected (black symbols) and preinfected (grey symbols) cells. Each symbol shows one individual experimental replicate. Data were pooled from three independent experiments. Horizontal bars denote the mean. ***p < 0.001; ns, not significant.

*L*. *major* has been shown to replicate once every 15 to 60 hours within host cells [18,22,30]. Thus, the high parasite proliferation observed in newly recruited host cells could be either due to the transmission of already rapidly proliferating parasites, or due to an increase of proliferation upon infection of a new host cell. In order to determine *L*. *major* proliferation shortly after transmission to new host cells, we employed in vitro cell culture infections. First, to visualize the spread of *L*. *major* to new host cells, we performed time-lapse microscopy of parasite uptake events into new host cells in macrophages cultured infected for 24 h. Strikingly, in all de novo infection events observed in this setting, fluorescent parasites were taken up directly from infected host cells into a new cell (**Fig 6A, S1 Movie**). Specifically, in most cases, the original host cells exhibited signs of cell death (membrane blebbing) shortly before the parasites associated with them were taken up by a new phagocyte (**Fig 6B**). Very often, the original host cell was eventually phagocytosed as well by the new host cell, however, transfer of the parasite preceded phagocytosis of the whole original host cell by several hours (**Fig 6C**). We therefore concluded that the infection of new host cells mainly occurs via cell-to-cell transmission from dying phagocytes. In order to quantitatively analyze pathogen proliferation shortly after infection of cells, we adapted our system for analyzing de novo infection (see **Fig 5C**) for in vitro cell culture infections using in vitro-differentiated bone marrow-derived dendritic cells and macrophages (**S4 Fig** Panel A). For this, we infected in vitro differentiated CD45.2^+^ bone marrow-derived macrophages (BMMP), dendritic cells (BMDC), or mixtures (BMMDC) with serum-opsonized *Lm*^SWITCH^. 24h after infection, the parasites were photoconverted and 24h later, CD45.1^+^ BMDC, BMMC, or BMMDC were added for 5 hours and the infection of newly added CD45.1^+^ cells was analyzed by flow cytometry (**Fig 6D**, **S4 Fig** Panels B-D). While the transition of parasites between preinfected non-mixed BMMP and BMDC varied dramatically in efficiency depending on the cell type used, we found that infection rates in BMMDC mixtures were much more homogenous (**S4 Fig** Panels C-E). Thus these mixtures were used for the analysis of newly infected phagocytes. Confocal microscopy of FACS-sorted newly infected CD45.1^+^ cells revealed that the parasites were intracellular and not extracellularly adhering to the cells (**Fig 6E**). Importantly, even at this very early time point after uptake into a new host cell, high proliferating parasites were significantly overrepresented in the newly infected CD45.1^+^ phagocytes as compared to the initially infected CD45.2^+^ cells (**Fig 6F and 6G, S4 Fig** Panel F-G). Control measurements with photoconversion applied just before co-culture ensured that the recovery from photoconversion during the 5h coculture phase was negligible (**Fig 6G, right**). Furthermore, both F4/80^+^, macrophage-like and CD11c^+^, dendritic cell-like BM-derived cells were infected equally by high proliferating parasites, irrespective of iNOS production in the culture, probably due to the short infection times (**S4 Fig** Panel H-I). Thus, the differences in *L*. *major* proliferation observed in the in vitro system are not attributable to the host cell type in this system, but rather to the dissemination among phagocytes.

**Fig 7:**
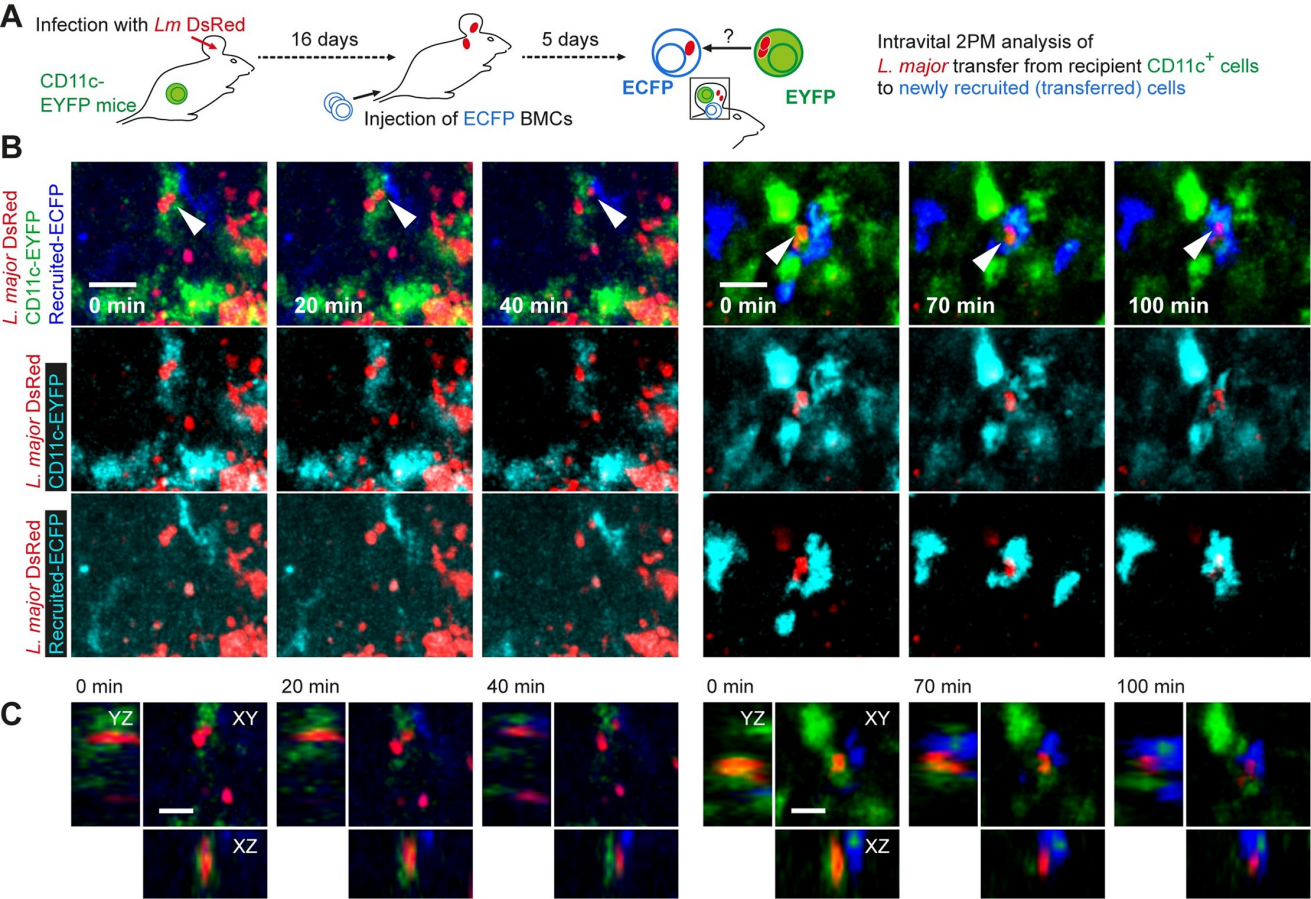
Intravital 2-photon microscopy demonstration of *L*. *major* cell-to-cell transfer from CD11c^+^ cells to newly recruited phagocytes. **(A)** Experimental setup for intravital 2-photon analysis of parasite cell-to-cell transfer. **(B)** Two examples of cell-to-cell transfer events observed in the ear of anesthetized mice. Images are selected projections of 10–15 slices of 3 μm-spaced z-stacks taken longitudinally every 10 minutes. Individual color overlays of DsRed (red) with host CD11c-EYFP and the ECFP expressed by newly recruited cells are shown separately in the middle and bottom line of the panel. Scale bar, 20 μm. **(C)** XYZ-sections showing single imaging planes (XY) or reconstructions (XZ, YZ) of the image stacks shown in (B). Scale bar, 10 μm.

In order to visualize the transfer of parasites from infected CD11c^+^ cells to newly recruited phagocytes in vivo, we infected CD11c-EYFP reporter mice with monofluorescent DsRed-expressing *L*. *major* for 16 days, adoptively transferred bone marrow cells from constitutively ECFP-expressing Actin-ECFP mice, and subjected the mice to intravital 2-photon imaging after five days (**Fig 7A**). We could observe the transit of DsRed-expressing parasites fully engulfed by (**Fig 7B and 7C, S2 Movie**) or juxtapositioned to (**S5 Fig**) CD11c-EYFP-expressing cells into newly recruited ECFP-expressing cells. This indicates cell-to-cell transfer of *L*. *major* from infected CD11c^+^ cells of the host to newly recruited monocytes in vivo. Of note, the transition occurred within about 1-2h, which corresponded very well with the observations from the in vitro system (see S1 Movie). Taken together, our data suggest that CD11c^+^ cells can harbor *L*. *major* which infect new host cells in vivo, and that high proliferating *L*. *major* preferentially undergo cell-to-cell transmission.

### *L*. *major* infects newly recruited monocytes at various differentiation stages

**Fig 8:**
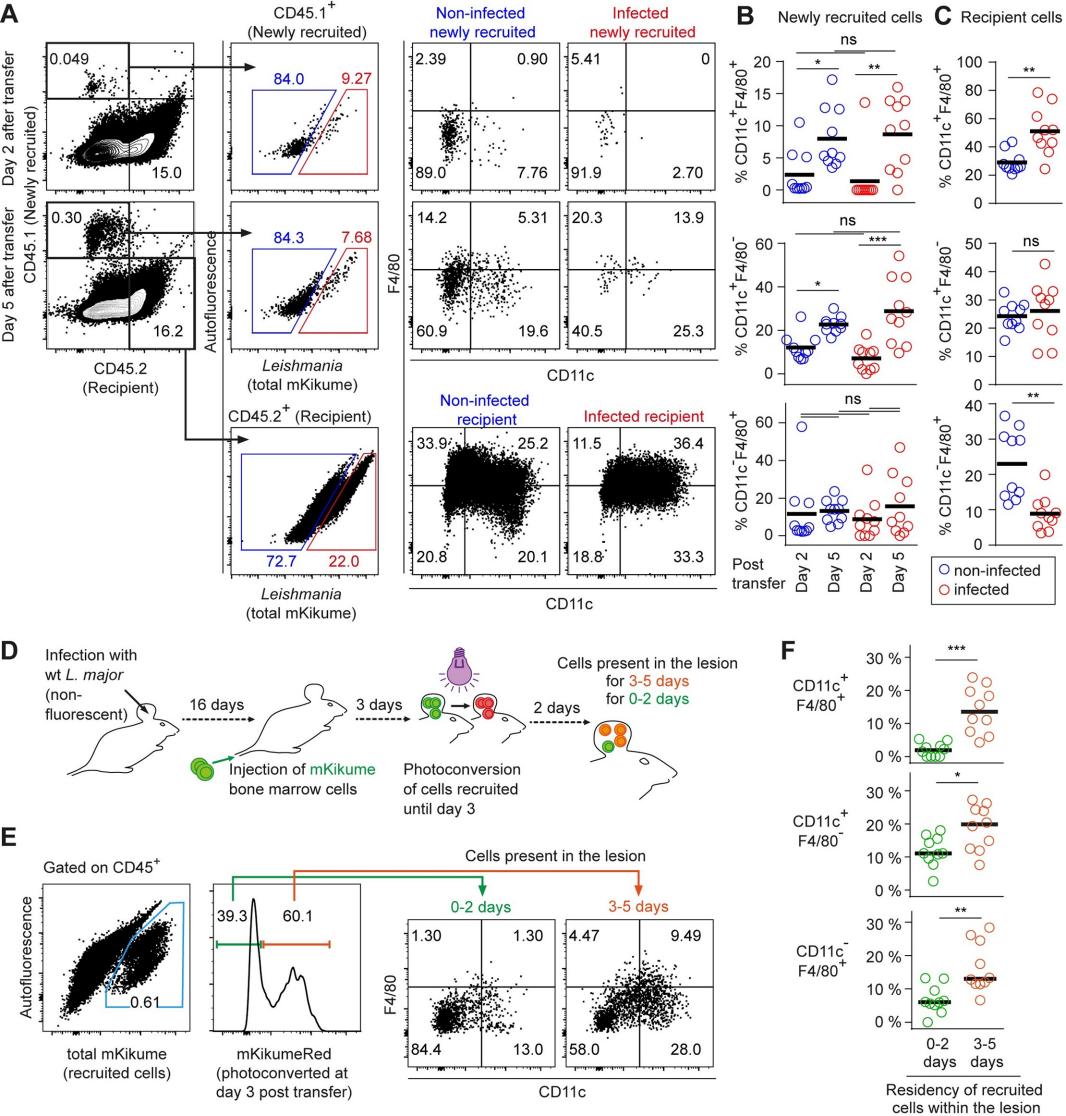
*L*. *major* infects newly recruited monocyte-derived cells independently of their differentiation stage. **(A)** Flow cytometry analysis of C57BL/6 (CD45.2) mice infected with *Lm*^SWITCH^, adoptively transferred 2 or 5 days before analysis with CD45.1 bone marrow cells. Gating strategy on CD45.1^+^ (newly recruited) and CD45.2^+^ (recipient) infected and non-infected cells. **(B)** Quantification of infected (red) and non-infected (blue) CD11c^+^ F4/80^+^ double positive (upper panel) and CD11c^+^ F4/80^-^ single positive (lower panel) among newly recruited cells at day 2 and day 5 post adoptive transfer. **(C)** Analysis as in (B) for recipient cells. **(D)** Experimental strategy to identify newly recruited cells present for 0–2 versus 3–5 days post adoptive transfer. Mice infected with non-fluorescent *L*. *major* wild type received mKikume-expressing bone marrow cells 5 days prior to analysis. Photoconversion at 3 days post transfer identifies cells recruited to the site of infection until day 3 in mKikume red fluorescence, while cells recruited after photoconversion (0–2 days prior to analysis) exhibit green mKikume fluorescence. **(E)** After gating on CD45^+^ cells, all adoptively transferred cells are identified by total mKikume fluorescence. Transferred cells which were at the infection site since 0–2 days (showing no red fluorescence) can be clearly distinguished from cells that were recruited to the site of infection 3–5 days before analysis (and thus photoconverted, showing a high red fluorescence). The two populations were analyzed for CD11c and F4/80 expression. **(F)** Quantitative analysis of CD11c^+^ F4/80^+^ double positive (upper panel) and CD11c^+^ F4/80^-^ single positive (lower panel) present at the site of infection for 0–2 days (green) and 3–5 days (red), respectively. Each dot represents one mouse ear. Horizontal bars denote the mean. *p < 0.05; **p < 0.01; ***p < 0.001; ns, not significant.

In order to investigate whether distinct subpopulations of newly recruited host phagocytes were preferentially infected upon arrival at the site of infection, we compared CD11c and F4/80 expression on infected and non-infected newly recruited cells (CD45.1^+^) and recipient cells (CD45.2^+^) expected to be, on average, present at the infection site for a longer time period before analysis (**Fig 8A–8C**). As expected from our previous work [33], the composition of the newly recruited phagocyte subsets changed substantially between day 2 and 5 after adoptive transfer. Specifically, CD11c^+^F4/80^+^ double positive as well as CD11c^+^F4/80^-^ cells were increased significantly by day 5 as compared to day 2 (**Fig 8B**). However strikingly, among newly recruited cells, no differences in the composition of infected versus non-infected cells were observed (**Fig 8B**, compare infected with non-infected). This suggests that the parasite has no preference for a specific cell type when infecting new host cells. In contrast, we found that in the recipient cell population, more CD11c^+^F4/80^+^ double positive and less CD11c^-^F4/80^+^ single positive phagocytes were present in the infected cell population as compared to the non-infected cells (**Fig 8C**). Thus, in line with our proliferation biosensor data (see **Figs 2 and 3**), CD11c^+^F4/80^+^ phagocytes seem to represent a more suitable niche for *L*. *major* in the long term perspective.

The late occurrence of CD11c^+^F4/80^+^ and CD11c^+^F4/80^-^ cells (**Fig 8B**) prompted us to determine how long the recruited monocyte-derived cells would need to mature to these populations at the site of infection [11]. In order to address this question, we infected C57BL/6 mice with non-fluorescent wild type *L*. *major* for 3 weeks and adoptively transferred mKikume-expressing bone marrow cells. 3 days after transfer, we marked by photoconversion all mKikume-expressing transferred cells that had already been recruited to the ear. Analysis of infected tissue at 5 days after transfer would also yield non-photoconverted mKikume-expressing cells, which consequently must have been recruited between day 3 and 5 (**Fig 8D and 8E**). This enabled us to compare the surface marker expression of cells present at the site of infection for longer (photoconverted), or shorter (not photoconverted) than two days. Control experiments showed that cell metabolism-related recovery from photoconversion in leukocytes is slow enough to use mKikume to mark cells over several days (**S6 Fig**). Importantly, we observed that the large majority of cells expressing CD11c and F4/80 were photoconverted, thus had been present in the lesion at least for 2 days. We concluded that these cells had matured at the site of infection from cells recruited between day 1 and 3 after transfer. In contrast, cells present at the site of infection for less than 3 days are in a substantially less mature state (**Fig 8E and 8F**). Therefore, these data underline that newly recruited monocyte-derived phagocytes are infected by *L*. *major* irrespective of their differentiation state.

## Discussion

To understand the interaction between the immune system and the pathogen, it is indispensable to extract data on the proliferation states of infectious microorganisms as well as to define the niches in which differentially proliferating populations are located. This information is especially critical for intracellular pathogens persisting for long periods of time at an infection site, such as *L*. *major*. Several recent approaches have addressed this question in the ongoing infection [22,30]. These elegant experiments have defined slow overall proliferation rates and postulated high proliferating subpopulations of parasites in the established infection, but neither approach was compatible with intravital or multiparameter host cell-resolved analysis of pathogen proliferation. This has severely hampered the unambiguous assignment of surface marker expression levels to individual host cells harboring high versus low proliferating parasites. However, this information would be indispensable for elucidating the link between *L*. *major* proliferation and host cell tropism. We achieved exactly this side-by-side analysis of pathogen proliferation and cellular surface markers by using the mKikume reporter system. Specifically, our experiments show that fluorescence recovery after photoconversion-based measurement of *Lm*^SWITCH^ infected cells and tissues is compatible not only with intravital 2-photon microscopy, but also with immunofluorescence-based analysis approaches such as flow cytometry as well as confocal and multiparameter microscopy (MELC).

In line with previous BrdU-based proliferation measurements [22], we show that *L*. *major* parasites grow at a broad distribution of different proliferation rates. The harsh fixation conditions in bioorthogonal labeling experiments preclude a concomitant analysis of the cellular niche of the pathogens. In contrast, we show that individual host cells contain parasites with a proliferation rate which is similar among all *L*. *major* within the same cell. Thus, we conclude that *L*. *major* proliferation rate is linked to the cellular niche in which the parasite resides.

In the established infection, this niche is constituted mainly of monocyte-derived phagocytes, many of them have been shown to express CD11c. Specifically, monocyte-derived dendritic cells have been proposed to mature in the skin after their recruitment, and then serve as important initiators for protective adaptive immune responses [11]. Furthermore, monocyte-derived iNOS and TNF-producing dendritic cells have been proposed to constitute a major fraction of *L*. *major*-infected cells and represent the main producers of iNOS in the skin [10], a defence mechanism critical for *L*. *major* containment. On the other hand, using cell type-specific gene ablation, it was shown that Interleukin-10 receptor signaling in CD11c^+^ cells is involved in dampening the immune response against *L*. *major* burden at the peak of the infection [16]. Furthermore, a recent study showed that maturation to a dendritic cell phenotype is not required for iNOS-production. Instead, Ly6C^+^CCR2^+^CX_3_CR1^+^ inflammatory monocytes were identified as important effector cells producing iNOS during secondary infections [12]. Whether any of these cell types would be particularly permissive for higher pathogen proliferation in vivo had remained uncharacterized.

Our multiparameter microscopy analysis approach suggested that CD11c^+^ expression by infected phagocytes correlates with high pathogen proliferation, irrespective of the level of F4/80, class II MHC and CD86. However, CD11c^+^F4/80^+^ cells seemed to be overrepresented among infected cells, thus representing an important niche for the parasite. We furthermore show that these cells also express high levels of CCR2 and Ly6C, thus suggesting that they have characteristics of inflammatory monocytes [12], but also of dendritic cells, as suggested by the high level of MHC class II. Based on the observation that CD11c^+^Ly6C^+^, but not CD11c^+^Ly6C^-^ cells harbor high proliferating *L*. *major*, we conclude that the cell population most permissive for high parasite proliferation has important phenotypic similarities with monocyte-derived dendritic cells [17,38].

Dendritic cells have been shown to harbor phagocytosed antigen at near-neutral pH, which has been proposed to ensure efficient antigen presentation [40]. In contrast, the intracellular amastigote form of *Leishmania* seems to preferentially proliferate at low pH for differentiation and proliferation [41]. Interestingly, the parasite can evade antigen presentation by decreasing the intraphagosomal pH within dendritic cells [42]. With regard to our finding that the niche of high-poliferating *L*. *major* has characteristics of monocyte derived dendritic cells, a parasite-induced pH decrease might, besides interference with antigen presentation, serve the generation of a parasitophorous vacuole which has a pH optimal for rapid proliferation. Alternatively, as different maturation stages towards monocyte-derived dendritic cells are present at the site of infection [11], it is possible that within these cells, the pH is lower than in mature dendritic cells after migration to the lymph node.

Our previous work had shown that several layers of cell-extrinsic *L*. *major* containment are in place. First, a gradient of IFN-γ mediates induction of iNOS, the main cellular defense mechanism against the parasite, also in cells that are not directly engaged by effector T cells [39]. Second, diffusible nitric oxide produced by iNOS seems to provide another layer of cooperative control of the pathogen on the tissue level [33]. In contrast to these findings, our data showing that proliferation rates are linked with a specific cell type suggest that additional cell-intrinsic control mechanisms against *L*. *major* proliferation exist. This could be achieved by differential production of reactive oxygen, which is generated through induction of the NADPH oxidase machinery at the phagosomal membrane in macrophages [43,44]. Nitric oxide, together with reactive oxygen, can form highly toxic peroxynitrite, which has a diffusion range of less than 5 μm from its site of production [45,46]. Thus, while nitric oxide seems to diffuse to neighboring cells, peroxynitrite formation at sites of high NADPH oxidase activity might represent a cell-intrinsic component of *L*. *major* containment. A further possible explanation for distinct proliferation rates within different cells might be the capacity of the parasites to counteract cellular defense mechanisms by deactivating NADPH oxidase assembly, detoxifying enzymes or interference with host phagocyte signaling pathways linked with antimicrobial activity, which might not be equally efficient in all cell types [47,48]. Finally, while a tissue-wide mode of *L*. *major* control could be mainly shown for CD4^+^ T cell-dependent effector functions, CD8^+^ cytotoxic T cells and NK cells might mediate target cell-intrinsic containment mechanisms [49–51].

Strategies of intracellularly proliferating pathogens to exit infected cells in order to be transmitted into a new cellular niche are critical for survival of pathogens especially in long-lasting infections [1]. This process has profound implications for the cell tropism of the pathogens, as well as immune activation, but has not been understood. In vitro evidence from *L*. *amazoniensis* suggests direct cell-to-cell transfer via LAMP1-rich extrusions [52]. Furthermore, intravital 2-photon imaging had shown that the *L*. *major* is taken up by neutrophils immediately after inoculation of the skin, and is then phagocytosed by both macrophage and dendritic cell-like phagocytes, a process that involves the apoptosis of the neutrophils [9,53]. While in the established infection later on, monocytes and monocyte-derived macrophages and dendritic cells have been shown to represent the main infected cell type [10–12], nothing is known about how *L*. *major* disseminates to new host cells during this phase. Our in vitro time-lapse microscopy suggests that apoptosis of the original host cell might also be involved during the dissemination from host cells different from neutrophils. Furthermore, synchronization of the arrival of newly recruited phagocytes revealed that high proliferating parasites are more efficiently transmitted to newly recruited host cells. In line with this, not only CD11c^+^Ly6C^+^ cells, but also some Ly6C^+^CD11c-negative phagocytes were observed to harbor high proliferating pathogen. We speculate that within this population, newly recruited monocytes are overrepresented, which we could show to harbor mainly high proliferating *L*. *major*. It is conceivable that eventually, the newly infected CD11c-negative monocyte-derived populations are able to dampen pathogen proliferation, resulting in the observed overall distribution of high proliferating parasites in CD11c-expressing monocytes and low proliferating parasites in most other host cell types.

The monocytes infiltrating the site of infection have been shown to coexist in a variety of maturation states, and *L*. *major* uptake can dampen the maturation of infected host cells [11,12]. By marking newly arrived monocytes at the site of infection, we could show that these cells require more than two days for differentiation at the site of infection. The potential of *L*. *major* to rather non-specifically infect different cell types at different maturation states, might therefore support the parasite’s ability to prevent efficient maturation of the recruited cells.

Of note, our in vitro data suggest that the high pathogen proliferation index detected in newly recruited cells is due to the successful transmission of *L*. *major* which were already exhibiting a higher proliferation rate, and not due to an increase in proliferation upon infection of a new host cell. We therefore propose that the CD11c-expressing monocytes not only represent a cellular niche of high *L*. *major* proliferation, but also the main source of parasites disseminating to new cells. In contrast, it is likely that CD11c-negative monocyte-derived cells, although initially infected as efficiently as the CD11c^+^ cells, fuel the cycle of intracellular proliferation, infection of new host cells, and thus the dissemination of the parasite in the infected skin, much less efficiently.

Our study focuses on the acute phase of the infection, with high pathogen burden and increasing pathology [39], in which we assume that efficient establishment of the parasite at the infection site is ensured by massive proliferation. Also, we have shown in an earlier study that non-lethal dampening of parasite proliferation with very little overt killing can efficiently contain the parasite in this phase [18]. Related to this, our demonstration of short-term depletion of CD11c^+^ cells resulting in a decreased parasite burden supports the hypothesis that CD11c-expressing monocytes can influence the pathogenesis of *L*. *major* also by serving as a niche for efficient establishment of an infection. However, as parasite replication represents a source of non-self antigen and pathogen-associated molecular pattern molecules, decreased proliferation is probably not purely detrimental for *L*. *major*. For example, it is possible that the establishment of a balance of low pathogen burden and low pathology, observed at very late phases of *L*. *major* infection [54], is achieved by residence within host cells that permit only low proliferation rates.

Taken together, besides their role in the maintenance of an adaptive immune response in a variety of infections [11,55,56], our findings establish that CD11c-expressing monocytes can represent a reservoir for rapidly proliferating *L*. *major* that disseminate at the site of infection. This quantification a pathogen physiology in the ongoing infection can critically contribute to our understanding of interactions between infectious organisms with the host immune system.

## Methods

### Ethics Statement

All animal experiments were reviewed and approved by the Ethics Committee of the Office for Veterinary Affairs of the State of Saxony-Anhalt, Germany (permit license numbers 42502-2-1253 Uni MD, and 42502-2-1314 Uni MD) in accordance with legislation of both the European Union (Council Directive 499 2010/63/EU) and the Federal Republic of Germany (according to § 8, Section 1 TierSchG, and TierSchVersV).

### Parasites and mouse infections

*L*. *major* LRC-L137 V121 wild-type, DsRed or mKikume expressing *Lm*^SWITCH^ parasites were previously described [18,57,58]. Parasites were grown in M119 medium completed with 10% heat-inactivated fetal calf serum, 0.1 mM adenine, 1 mg/ml biotin, 5 mg/ml hemin, and 2 mg/ml biopterin (all from Sigma) for maximally 6 passages.

Wild-type CD45.1 (B6.SJL-*Ptprc*^*a*^*Pepc*^*b*^/BoyJ), Actin-ECFP (B6.129(ICR)-Tg(CAG-ECFP)CK6Nagy/J), CD11c-EYFP (B6.Cg-Tg(Itgax-Venus)1Mnz/J), CD11c-DTR-GFP^tg^ (B6.FVB-Tg(Itgax-DTR/EGFP)57Lan/J) and mKikume expressing (Tg(CAG-KikGR)33Hadj/J) mice were purchased from Jackson Laboratories (Bar Harbor, MA), wild-type C57BL/6J and B6N-Tyrc BrdCrCrl (B6 albino wild-type) mice were obtained from Charles River (Sulzfeld, Germany). All mice were bred under specific pathogen-free conditions at Otto-von Guericke University, Magdeburg. For the infection of mice, stationary phase parasites were centrifuged (3500 g, 5 min, RT) and resuspended in PBS. 2x10^6^ parasites were subsequently injected in 10 μl into the ear dermis. Analysis was performed 3 weeks post infection.

### Intravital imaging

Mice were anaesthetized and prepared for intravital microscopy as described previously [39]. Two-photon imaging was performed with a W Plan-Apochromat 20x/1,0 DIC VIS-IR objective (Zeiss) on a LSM 700 confocal laser scanning microscope (Zeiss) and a Mai Tai DeepSee laser (Spectra-Physics) tuned at 920 nm. For analysis of parasite proliferation in vivo, the emitted mKikume signal and second harmonics were split with 625 nm long pass, 495 nm long pass, and 555 nm long pass dichroic mirrors and filtered with 470/20 (second harmonics), 525/50 (mKikume green) and 600/40 (mKikume red) nm bandpass filters before collection with nondescanned detectors. For intravital analysis of cell-to-cell transmission, ECFP, EYFP and DsRed fluorescence as well as harmonics were split with 560 nm long pass, 470 nm long pass, and 520 nm long pass dichroic mirrors and filtered with 600/40 (DsRed), 470/20 (second harmonics), 506/20 (ECFP) and 543/20 (EYFP) nm bandpass filters. Typically, imaging volumes of 0.8 mm^3^ for automated analysis were obtained by collecting 3–4 μm spaced z stacks using the ZEN acquisition software (Zeiss). Images were color corrected using the channel arithmetics function, superimposed and analyzed using the Imaris software (Bitplane), 3D projections and slices were extracted using the Fiji software (NIH, http://rsb.info.nih.gov/ij/).

### Photoconversion

*Lm*^SWITCH^ parasites in the mouse ear were photoconverted with violet light at 405 nm wavelength by assembling 2x2 LED diodes (Strato, half-viewing angle: 15°; Radiant Power: 10 mW) spaced 5 mm apart. Ears of anaesthetized mice were fixed and illuminated from each side for 1 minute in a distance of 1.3 cm. The photoconverted parasites were analyzed after 48h by flow cytometry, multi-epitope ligand cartography, confocal microscopy or intravital microscopy. For in vitro analysis of de novo infection, parasites were photoconverted in 24-well plates via illumination with 405 nm wavelength by assembling 3x3 diode (see above) array for 1 minute and analyzed after 24h via flow cytometry.

### Multi-Epitope Ligand Cartography (MELC)

Ears were harvested and incubated for 2 h at 4°C in 4% paraformaldehyde in phosphate-buffered saline before they were stored in 20% sucrose in phosphate-buffered saline at 4°C overnight. Samples were frozen in Tissue-Tek O.C.T. Compound (Sakura) by liquid nitrogen and stored at -80°C. 10 μm cryosections were transferred on a 0.1% Poly-L-Lysin (Sigma-Aldrich) in H_2_O coated Superfrost slides (Thermo Scientific) and air-dried. Multi-Epitope Ligand Cartography was performed as previously described [34]. In brief, directly labelled antibodies (**S2 Table**) were incubated consecutively and 3D images of the fluorescence signal were acquired by a DMI6000B microscope (Leica) equipped with a 40x/NA1.25 lens and a KX4 CCD camera (Apogee Instruments) resulting in 3D image stacks of 2048 × 2048 × 16 or 8 voxels (voxel size 225 × 225 × 500 or 1000 nm^3^). The fluorescence signals were removed by bleaching of the directly coupled fluorophores. Using the corresponding phase contrast images acquired with every staining cycle, the fluorescence images were automatically aligned voxel-wise with accuracy of 1/10 pixel in XY direction and ½ pixel in Z direction. Illumination faults of the fluorescence images were eliminated using flat-field correction before the resolution of the wide field fluorescent image stacks were improved by applying a deconvolution/deblurring algorithm (XCOSM software package), an interface to Computational Optical Sectioning Microscopy algorithms for removing out-of-focus light in 3D image volumes (Washington University St. Louis, MO).

### Flow cytometry

Ears of mice were separated in two sheets (ventral and dorsal) using forceps and enzymatically digested in RPMI 1640 medium containing 1 mg/ml collagenase (Sigma) and 50 ng/ml DNase (Sigma-Aldrich) for 60 min at 600 rpm and 37°C, and passed through a 70 μm cell strainer. Surface staining of cells was done by using APC or APC-Cy7 conjugated anti-CD45.2 (clone 104), APC, PerCP-Cy5.5 or APC-Cy7 conjugated anti-CD45.1 (clone A20), BV421 conjugated anti-F4/80 (clone BM8), Pe-Cy7, APC or APC-Fire conjugated anti-CD11c (clone N418), PE-Cy7 or APC-Cy7 conjugated anti-Ly6C (clone HK1.4), FITC conjugated anti-CCR2 (clone SA203G11), BV510 conjugated anti-MHC class II (IA/IE, clone M5/114.15.2), APC or APC-Cy7 conjugated anti-CD11b (clone M1/70), and PerCP-Cy5.5 conjugated anti CD45 (clone 30-F11), which were all purchased from BioLegend. Samples were Fc-blocked using anti-CD16/32 antibody (clone 93) (BioLegend) before antibody staining. Analysis was performed with a Fortessa or FACS ARIA III (BD Biosciences) using 405, 488, 561, and 633 nm lasers:. Photoconverted or non-photoconverted mKikume fluorescence was read out at 561 nm excitation and 610/20 nm emission, or 488 nm excitation and 530/30 nm emission, respectively. An autofluorescence signal was recorded at 488 excitation and 695/40 nm emission. Data were analyzed by using the FlowJo X software (FlowJo, LLC).

### Confocal microscopy

Ears were harvested and incubated for 2 h at 4°C in 4% paraformaldehyde in phosphate-buffered saline before incubation in 20% sucrose in phosphate-buffered saline at 4°C overnight. Samples were frozen in Tissue-Tek O.C.T. Compound (Sakura) in liquid nitrogen and stored at -80°C. 10 μm cryosections were prepared, transferred onto Poly-L-Lysin (0.1% in H_2_O for coating) coated Superfrost slides (Thermo Scientific), air-dried and stained with Armenian hamster anti-CD54 (clone 3E2, from BD Biosciences) and DyLight649-conjugated goat anti-Armenian hamster Ig (Jackson ImmunoResearch). Analysis was performed by confocal laser scanning microscopy (TCS SP8 Confocal, Leica). 488 nm excitation and 491–526 nm emission was used for non-photoconverted mKikume, 561 nm excitation and 571–620 nm emission for photoconverted mKikume, and 633 nm excitation and 640–720 nm for detection of the CD54 staining. Image analysis was done with the Fiji software (NIH, http://rsb.info.nih.gov/ij/).

### In situ cytometry analysis

Deconvolved MELC image stacks of CD45, CD54, and CD11b stainings were segmented using the RACE tool developed by Stegmaier et al. [35]. The Propidium Iodide staining was used as nuclei seed dataset. In brief, the segmented images were converted into ImageJ regions of interest (ROIs) for three Z planes spaced 3 μm apart and centered around the middle of the image stack, and combined for the three markers. RACE parameters were optimized in order to detect the largest number of cells and highest percentage of infected cells (see S1 Text and S1 Table for a detailed description of the optimization and segmentation procedure). Mean fluorescence values of the cell area as well as a 0.4 μm rim zone were extracted, normalized between the 20^th^ and 80^th^ percentile of the corresponding fluorescence values of each image, and converted into .fcs FlowJo files using the DiscIT software [36]. Thresholds for individual markers were set according to at least 30 marker-positive and 30 marker-negative cells manually selected in three different images (**S2 Fig** Panel E).

### Proliferation analysis

The relative proliferation index of *L*. *major* within the different cell populations for both flow cytometry and MELC was defined as

$$1-\frac{{\left(\frac{mKikume\;Red}{mKikumeGreen}\right)}_{cell}}{{\left(\frac{mKikumeRed}{mKikumeGreen}\right)}_{mean\;(all\;infected\;cells)}}$$

and represented as percent deviation from the total infected cell population within one sample or imaged infection site. For visualizing qualitative comparisons within the same sample using the FlowJo software, values were plotted as

$$C-\frac{100*{\left(\frac{mKikume\;Red}{mKikumeGreen}\right)}_{cell}}{{\left(\frac{mKikumeRed}{mKikumeGreen}\right)}_{mean\;(all\;infected\;cells)}}$$

with chosen C between 100 and 250 and kept constant within the same sample for which the comparison was made, and the factor 100 introduced in order to analyze integer fluorescence values in FlowJo.

For in vitro determination and inter-experiment standardization of proliferation indices, a non-photoconverted (green control) and fully photoconverted (0h recovery from photoconversion, red control) sample were measured with each experiment, and for each infected cell, the proliferation index was defined as

$$10-\frac{100*{\left(\frac{mKikume\;Red}{mKikumeGreen}\right)}_{cell}-100*{\left(\frac{mKikume\;Red}{mKikumeGreen}\right)}_{mean\;(all\;infected\;cells,\;green\;control)}}{100*{\left(\frac{mKikume\;Red}{mKikumeGreen}\right)}_{mean\;(all\;infected\;cells,\;red\;control)}-100*{\left(\frac{mKikume\;Red}{mKikumeGreen}\right)}_{mean\;(all\;infected\;cells,\;green\;control)}}$$

with the factor 100 introduced in order to analyze integer fluorescence values in FlowJo, and the constant 10 in order to obtain positive values.

### Bone marrow isolation and cell transfer

Bone marrow cells were isolated from tibia and femur of mice and passed through a 70 μm cell strainer. 8–10 x 10^7^ cells were resuspended in 300 μl PBS and intravenously injected into the recipient mice 2 or 5 days before the analysis.

### Cell culture infections

Bone marrow cells from either C57BL/6 or CD45.1 wildtype mice were filtered through a 70 μm cell strainer in PBS before they were differentiated in vitro into macrophages and dendritic cells. For differentiation of macrophages, cells were plated in RPMI 1640 (Merck) supplemented with 10% FCS and 20% 3T3 cell culture supernatant and incubated at 37°C and 5% CO_2_. Three days later, the medium was exchanged for fresh medium and after another four days of incubation macrophages were used for infection experiments. Dendritic cells were differentiated by culturing isolated bone marrow cells in BM-DC medium (1x NEAA (Gibco), 5% FCS (PAA), 2 mM L-Gltamin (Gibco), 50 μM b-Mercaptoethanol (Gibco), 50 μg/ml Genatmycin (Gibco), 100 U/ml IL-4, 255 U/ml GM-CSF at 37°C and 5% CO_2_ for three days. Afterwards, the medium was exchanged for fresh medium and after another four days of incubation the dendritic cells were used for infection experiments.

For infection, differentiated macrophages and dendritic cells from C57BL/6 mice (CD45.2) were pooled in a ratio of 1:1 and *Lm*^SWITCH^ stationary phase promastigotes (opsonized with 5% mouse immune serum for 30 min at 26°C) were added with a MOI of 5. 24h later, parasites were photoconverted. Cells were induced with IFN-gamma (0.01 ng/μl, R&D Systems) and LPS (1μg/ml, E. coli O26:B6, Sigma-Aldrich) and optionally, the nitric oxide synthase iNOS was inhibited by addition of N6-(1-iminoethyl)-L-lysine hydrochloride (L-NIL) (0.023μg/μl, Sigma-Aldrich). After another 24h, a 1:1 mixture of CD45.1 macrophages and dendritic cells was added to the cell culture. After 5h of coculture, cells were analyzed by flow cytometry. For isolation of peritoneal macrophages mice were sacrificed and subsequently 5 ml of cold PBS were injected intraperitoneally. The cell suspension was aspirated and cells were seeded in RPMI 1640 supplemented with 10% heat-inactivated fetal bovine serum for infection and live cell imaging.

Time-lapse microscopy of cell culture infections was performed with the use of a DMI6000B inverted microscope (Leica Microsystems) or a CellR imaging workstation (Olympus) using an upright microscope stage (BX61) equipped with a 20x dry objectives. Images were automatically acquired every 10 minutes and movies were processed with the Fiji software (NIH, http://rsb.info.nih.gov/ij/).

### Statistical analysis

Spearman correlations and all comparisons between groups were calculated using the Prism 7 software (GraphPad Inc.). Statistical analysis of multiple cell populations was performed with by one-way analysis of variance (ANOVA) with a Tukey post-test for multiple cross-comparisons, and a Bonferroni post-test for comparison of selected pairs of conditions or with a control condition, respectively. Comparisons with only two experimental conditions were performed using a Mann Whitney test. P values under 0.05 were regarded as significant and marked with an asterisk. P values lower than 0.01 or 0.001 were hence allocated two or three asterisks, respectively.

## Supporting information

S1 Fig Multiparameter microscopy analysis strategy. **(A)** Example MELC images of different aligned fluorescence channels of an infected tissue site. A nonspecific IgG staining of the same site is shown for comparison. **(B)** Deconvolved and aligned image stacks of CD11b, CD45 and CD54 stainings were used separately as membrane input, the corresponding propidium iodide staining served as seed input for the Real-time Accurate Cell-shape Extractor (RACE) program. **(C)** From the resulting image stacks, regions of interest (ROIs) were generated. The cellular ROIs were used for mKikume fluorescence measurement. Rim masks generated from the ROIs were used for surface marker measurements. The mean fluorescence of the cellular and rim ROIs were extracted for each cell and fluorescence channel. **(D)** Definition of a threshold based on the total mKikume signal from infected and non-infected cells manually identified from three Z-planes in three independent experiments (each symbol represents one cell). **(E)** Upper panels, for defining fluorescence thresholds for gating within FlowJo, 30 marker-positive and 30 marker-negative cells were selected from images of three different sites of infection and a cutoff was defined (i.e. no marker-negative cells in the positive gate). Lower panels, examples of MELC datasets gated for marker-positive (green) and marker-negative (blue) infected cells. **(F)** Top row, marker positive (green gate) and marker negative (blue gate) cells were defined for each surface marker. Bottom row, proliferation rates of *Lm*^SWITCH^ in cell populations positive for CD45R, CD11c, F4/80, CD86 or MHCII (green histograms) were compared with the corresponding marker-negative cell populations (blue histograms). **(G)** Quantitative analysis of 7 individual MELC experiments performed in 4 different infected mice. Each symbol represents one imaged infection site, *p < 0.05.
(TIF)

S2 Fig Grid control of the flow-cytometry based proliferation analysis approach. **(A)** Grid illumination approach for scrambled photoconversion. Left panel, illumination grid (transmitted light image). Middle panel, intravital 2-photon image of a scramble-photoswitched *Lm*^SWITCH^-infected site in the ear, an image aligned to the grid showing photoswitched 80 μm spanning (dotted lines) parasite regions are shown. Right image: mKikume red channel only. A Z-projection of a 40 μm stack is shown in the middle and lower panel. Scale bars, 200 μm. **(B-C)** Experiment performed as described in Fig 3A–3D, but using arbitrarily photoconverted *L*. *major* using a grid.
(TIF)

S3 Fig Synchronization of newly recruited cell arrival. **(A)** Flow cytometry analysis of CD45.1 mice infected with *Lm*^SWITCH^, adoptively transferred 2 or 5 days before analysis with C57BL/6 (CD45.2) bone marrow cells. Gating strategy on CD45.2^+^ (newly recruited) and CD45.1^+^ (steady state) cells, infected and non-infected. **(B)** Quantification of newly recruited infected and non-infected cells at day 2 and day 5 post adoptive transfer. Each dot represents one mouse ear. Data shown are re-analysed from Fig 6. **(C-D)** C57BL/6 (CD45.2) mice were infected with *Lm*^SWITCH^ and CD45.1 bone marrow cells were adoptively transferred 5 days prior to analysis. Photoconversion was performed 48 h prior to analysis and the proliferation rates of parasites in steady state (CD45.2^+^ cells) and newly recruited (CD45.1^+^ cells) cells were compared. Antibody labels were switched for the anti-CD45.1 and anti-CD45.2 staining as compared to Fig 4. **(C)** Gating strategy for detection of infected CD45.1^+^ newly recruited and CD45.2^+^ steady state infected cells isolated from the site of infection (left and middle panel). Proliferation rates of the infected newly recruited and steady state population of one example site of infection are shown as histogram (right panel). Vertical bars denote the mean, and shaded red boxes the standard deviation. **(D)** Quantitative analysis of the proliferative state of *Lm*^SWITCH^ within the different cell types. Relative proliferation rates in newly recruited or steady state cells were obtained by normalization of the subpopulation’s proliferation indices to the overall mean proliferation index within each sample. Each dot represents one mouse ear. Vertical bars denote the mean. *p < 0.05.
(TIF)

S4 Fig A quantitative in vitro analysis approach for parasite cell-to-cell transfer. **(A)** Analysis of expression levels of uninfected CD11c, F4/80 and MHC class II in bone marrow-derived dendritic cell (green histograms) and macrophage (blue histograms) cultured. Data are representative of two independent experiments. **(B)** Analysis of CD11c versus F4/80 expression in infected dendritic cell (DC, left panel) and macrophage (MΦ, middle panel) and DC and MΦ mixed cultures (right panel). **(C)** Analysis of cell-to cell transfer efficiency from preinfected CD45.1^-^ bone marrow-derived dendritic cells (BMDCs) into CD45.1^+^ bone marrow-derived macrophages (BMMPs) (left part) or CD45.1^-^ BMMPs into CD45.1^+^ BMDCs (right part). **(D)** Analysis of cell-to cell transfer efficiency between mixed cultures of BMDCs and BMMPs (BMMPDC). **(E)** Quantitative comparison of infection rates in BMMCs (left graph) and BMDCs (right graph) as solely preinfected or newly recruited cells and in BMMPDC mixtures. Each symbol shows one individual experimental replicate. Data are pooled from two independent experiments. **(F)** Colour-switch control for *L*. *major* proliferation analysis in newly recruited cells, data shown are representative of three independent replicates **(G)** Quantification of *L*. *major* proliferation rates in newly infected (CD45.2^-^) and initially infected (CD45.2^+^) cells. Each symbol shows one individual experimental replicate. **(H)** Analysis of parasite proliferation in newly infected and initially infected cells under inhibition of the nitric oxide synthase iNOS by L-NIL and **(I)** in initially infected cells without inhibition of iNOS. ***p < 0.001; **p < 0.01; *p < 0.05; ns, not significant. Each symbol shows one individual experimental replicate.
(TIF)

S5 Fig Intravital 2-photon microscopy demonstration of de novo infection of newly recruited phagocytes by *L*. *major* juxtapositioned to a CD11c^+^ cell. **(A)** Two examples de novo infection experiments of newly recruited cells (blue) by *L*. *major* (red) initially juxtapositioned to a CD11c^+^ host cell (green). Images are selected projections of 10–13 slices of 3 μm-spaced z-stacks taken longitudinally every 10 minutes. Individual color overlays of DsRed (red) with host CD11c-EYFP and the ECFP expressed by newly recruited cells are shown separately in the middle and bottom line of the panel. Scale bar, 20 μm. **(B)** XYZ-sections showing single imaging planes (XY) or reconstructions (XZ, YZ) of the image stacks shown in (B). Scale bar, 10 μm.
(TIF)

S6 Fig mKikume expression in BM cells allows identification of photoconverted phagocytes after 48h of photoconversion. Ubiquitous mKikume expressing mice were infected with non-fluorescent *L*. *major* wild type. Photoconversion in the mouse ear was performed 48h prior to analysis. Control samples were photoconverted 0 h prior to analysis or not photoconverted at all. After gating on CD45^+^ cells, mKikume^+^ cells were identified. Cells which were photoconverted at the infection site 48h prior to analysis showed only a slight shift towards less red mKikume fluorescence, whereas non-photoconverted cells are recruited within this time period, indicating that metabolism-related recovery from photoconversion in mouse cells is not sufficient interfere with the identification of non-photoconverted, newly recruited cells.
(TIF)

S1 Table Optimization of RACE conditions for single cell detection. Deconvolved 400 x 400 x 8 micron stacks were segmented with the RACE settings indicated. Three infection sites from different mice (Site1-Site3) and two Z planes per site (ZPl1-ZPl2) were converted into flow cytometry datasets and analyzed as described in the supplementary methods (see S1 Text). The number of total and infected cells detected at each site/plane is indicated in the upper part of the table, the rank within one plane and site is shown in the lower part. The optimized condition is boxed.
(DOCX)

S2 Table Antibodies used for MELC. (DOCX)

S1 Text Supplementary methods. (DOCX)

S1 Movie Time lapse videomicroscopy of intraperitoneal macrophages infected for 24 h with fluorescently labeled *L*. *major*. Three examples of cell-to-cell transfer between two phagocytes are shown.
(MOV)

S2 Movie Intravital 2-photon microscopy of cell-to-cell transfer of *L*. *major* (red) from recipient CD11c-EYFP cells (green) into newly recruited adoptively transferred cells (blue). CD11c-EYFP^tg^ mice were infected in the ear for 16 weeks with monofluorescent *L*. *major* DsRed, ECFP-expressing bome marrow cells were adoptively transferred and the site of infection was images 5 days after transfer. Projections of 10–15 slices of 3μm-spaced z-stacks are shown.
(MOV)
