# Supplementary figures and images for: CD11c-expressing Ly6C+CCR2+ monocytes constitute a reservoir for efficient Leishmania proliferation and cell-to-cell transmission
Source: PLoS Pathog. 2018 Oct 22;14(10):e1007374. doi: 10.1371/journal.ppat.1007374 (PMC6211768; doi:10.1371/journal.ppat.1007374)

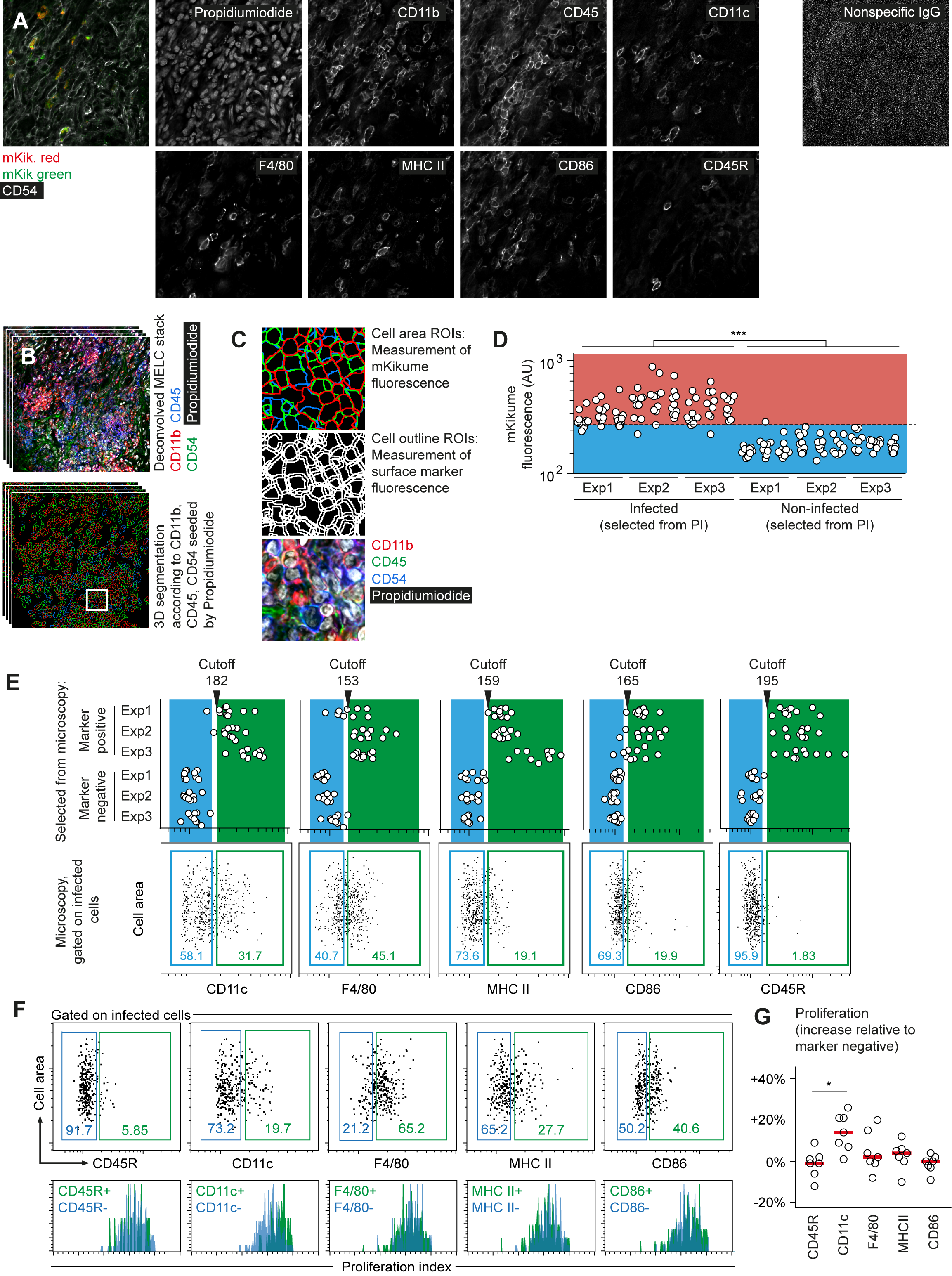

Supplement: S1 Fig — (A) Example MELC images of different aligned fluorescence channels of an infected tissue site. A nonspecific IgG staining of the same site is shown for comparison. (B) Deconvolved and aligned image stacks of CD11b, CD45 and CD54 stainings were used separately as membrane input, the corresponding propidium iodide staining served as seed input for the Real-time Accurate Cell-shape Extractor (RACE) program. (C) From the resulting image stacks, regions of interest (ROIs) were generated. The cellular ROIs were used for mKikume fluorescence measurement. Rim masks generated from the ROIs were used for surface marker measurements. The mean fluorescence of the cellular and rim ROIs were extracted for each cell and fluorescence channel. (D) Definition of a threshold based on the total mKikume signal from infected and non-infected cells manually identified from three Z-planes in three independent experiments (each symbol represents one cell). (E) Upper panels, for defining fluorescence thresholds for gating within FlowJo, 30 marker-positive and 30 marker-negative cells were selected from images of three different sites of infection and a cutoff was defined (i.e. no marker-negative cells in the positive gate). Lower panels, examples of MELC datasets gated for marker-positive (green) and marker-negative (blue) infected cells. (F) Top row, marker positive (green gate) and marker negative (blue gate) cells were defined for each surface marker. Bottom row, proliferation rates of LmSWITCH in cell populations positive for CD45R, CD11c, F4/80, CD86 or MHCII (green histograms) were compared with the corresponding marker-negative cell populations (blue histograms). (G) Quantitative analysis of 7 individual MELC experiments performed in 4 different infected mice. Each symbol represents one imaged infection site, *p < 0.05. (TIF) [file ppat.1007374.s001.tif]

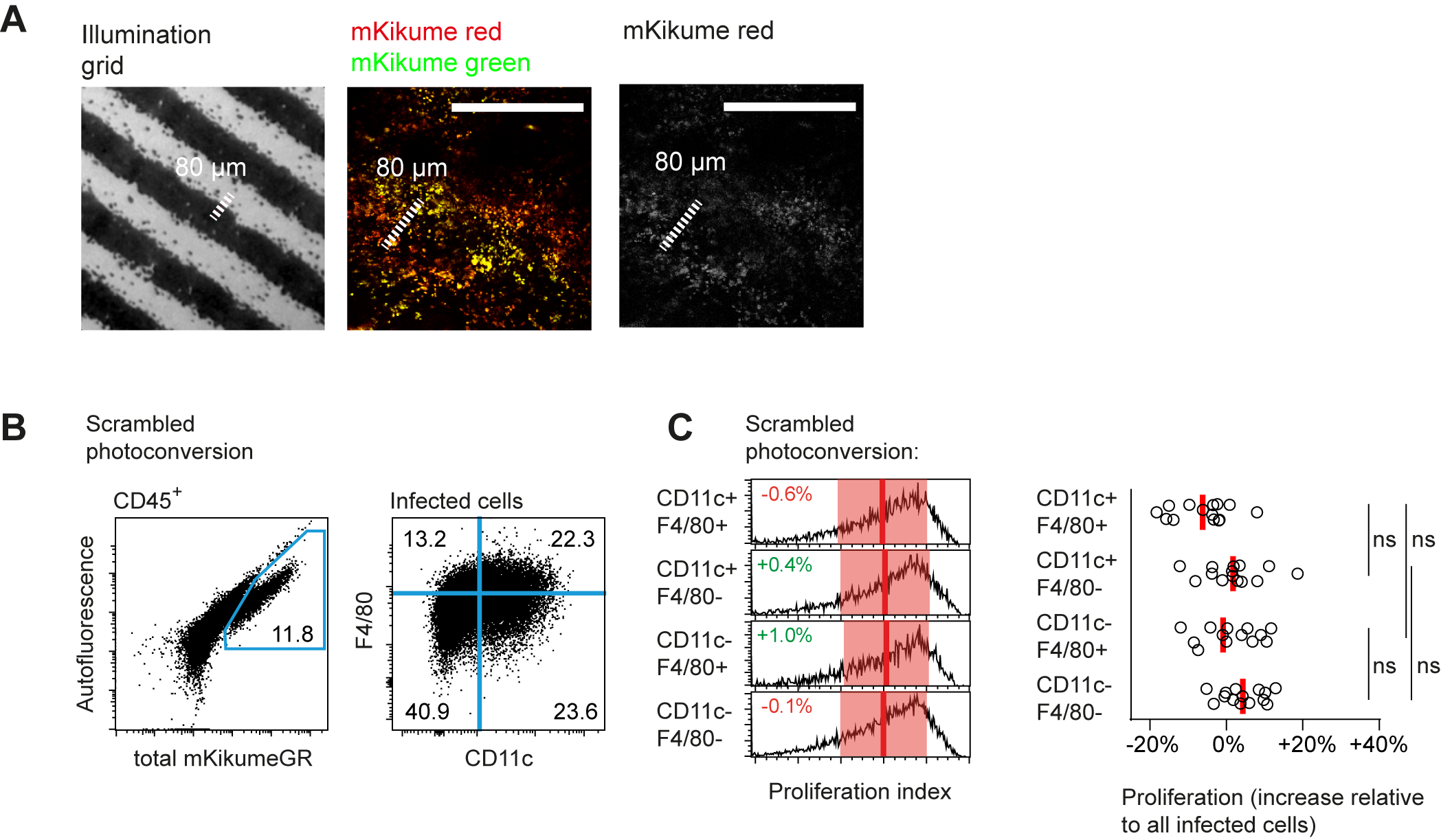

Supplement: S2 Fig — (A) Grid illumination approach for scrambled photoconversion. Left panel, illumination grid (transmitted light image). Middle panel, intravital 2-photon image of a scramble-photoswitched LmSWITCH-infected site in the ear, an image aligned to the grid showing photoswitched 80 μm spanning (dotted lines) parasite regions are shown. Right image: mKikume red channel only. A Z-projection of a 40 μm stack is shown in the middle and lower panel. Scale bars, 200 μm. (B-C) Experiment performed as described in Fig 3A–3D, but using arbitrarily photoconverted L. major using a grid. (TIF) [file ppat.1007374.s002.tif]

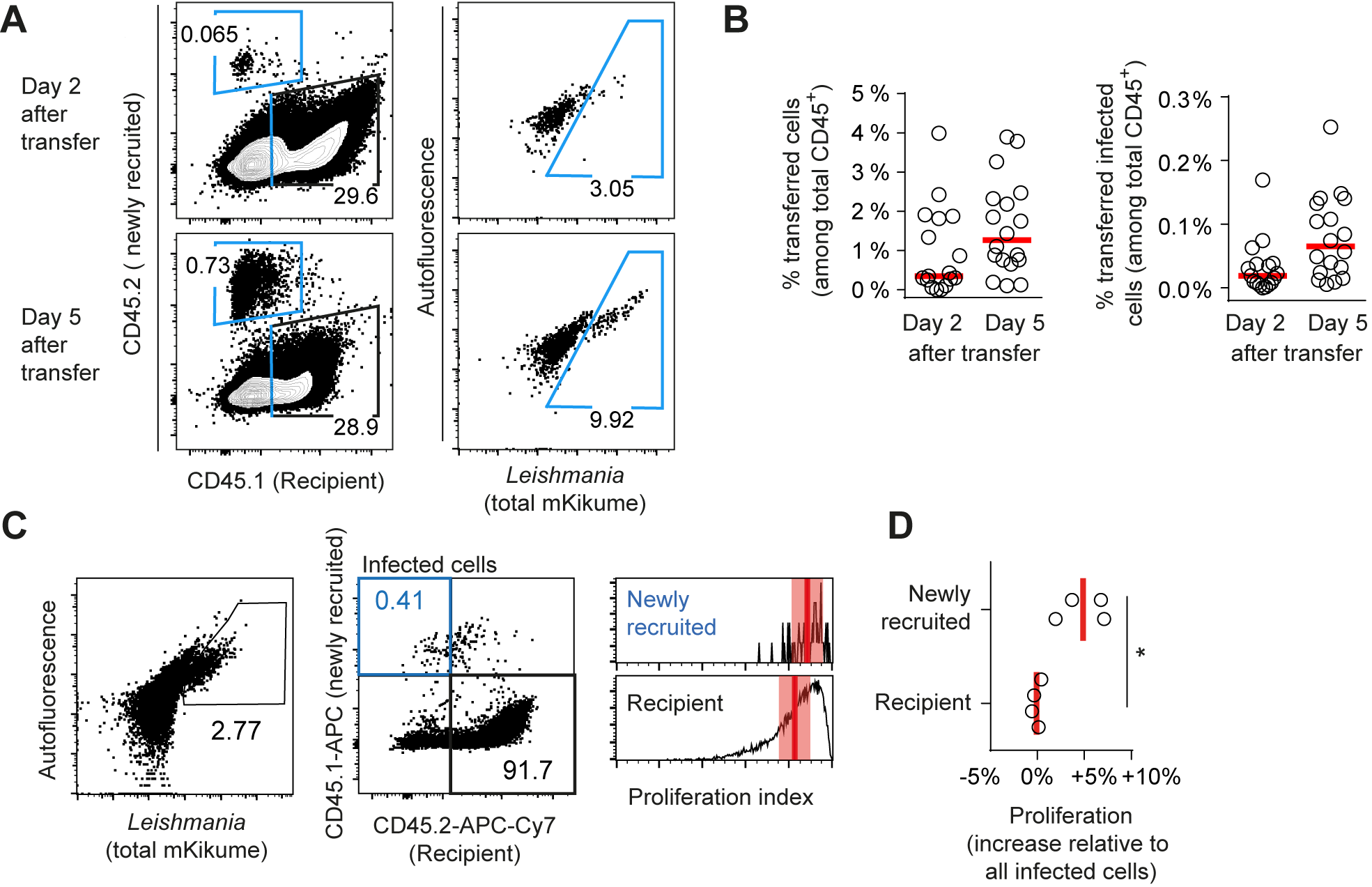

Supplement: S3 Fig — (A) Flow cytometry analysis of CD45.1 mice infected with LmSWITCH, adoptively transferred 2 or 5 days before analysis with C57BL/6 (CD45.2) bone marrow cells. Gating strategy on CD45.2+ (newly recruited) and CD45.1+ (steady state) cells, infected and non-infected. (B) Quantification of newly recruited infected and non-infected cells at day 2 and day 5 post adoptive transfer. Each dot represents one mouse ear. Data shown are re-analysed from Fig 6. (C-D) C57BL/6 (CD45.2) mice were infected with LmSWITCH and CD45.1 bone marrow cells were adoptively transferred 5 days prior to analysis. Photoconversion was performed 48 h prior to analysis and the proliferation rates of parasites in steady state (CD45.2+ cells) and newly recruited (CD45.1+ cells) cells were compared. Antibody labels were switched for the anti-CD45.1 and anti-CD45.2 staining as compared to Fig 4. (C) Gating strategy for detection of infected CD45.1+ newly recruited and CD45.2+ steady state infected cells isolated from the site of infection (left and middle panel). Proliferation rates of the infected newly recruited and steady state population of one example site of infection are shown as histogram (right panel). Vertical bars denote the mean, and shaded red boxes the standard deviation. (D) Quantitative analysis of the proliferative state of LmSWITCH within the different cell types. Relative proliferation rates in newly recruited or steady state cells were obtained by normalization of the subpopulation’s proliferation indices to the overall mean proliferation index within each sample. Each dot represents one mouse ear. Vertical bars denote the mean. *p < 0.05. (TIF) [file ppat.1007374.s003.tif]

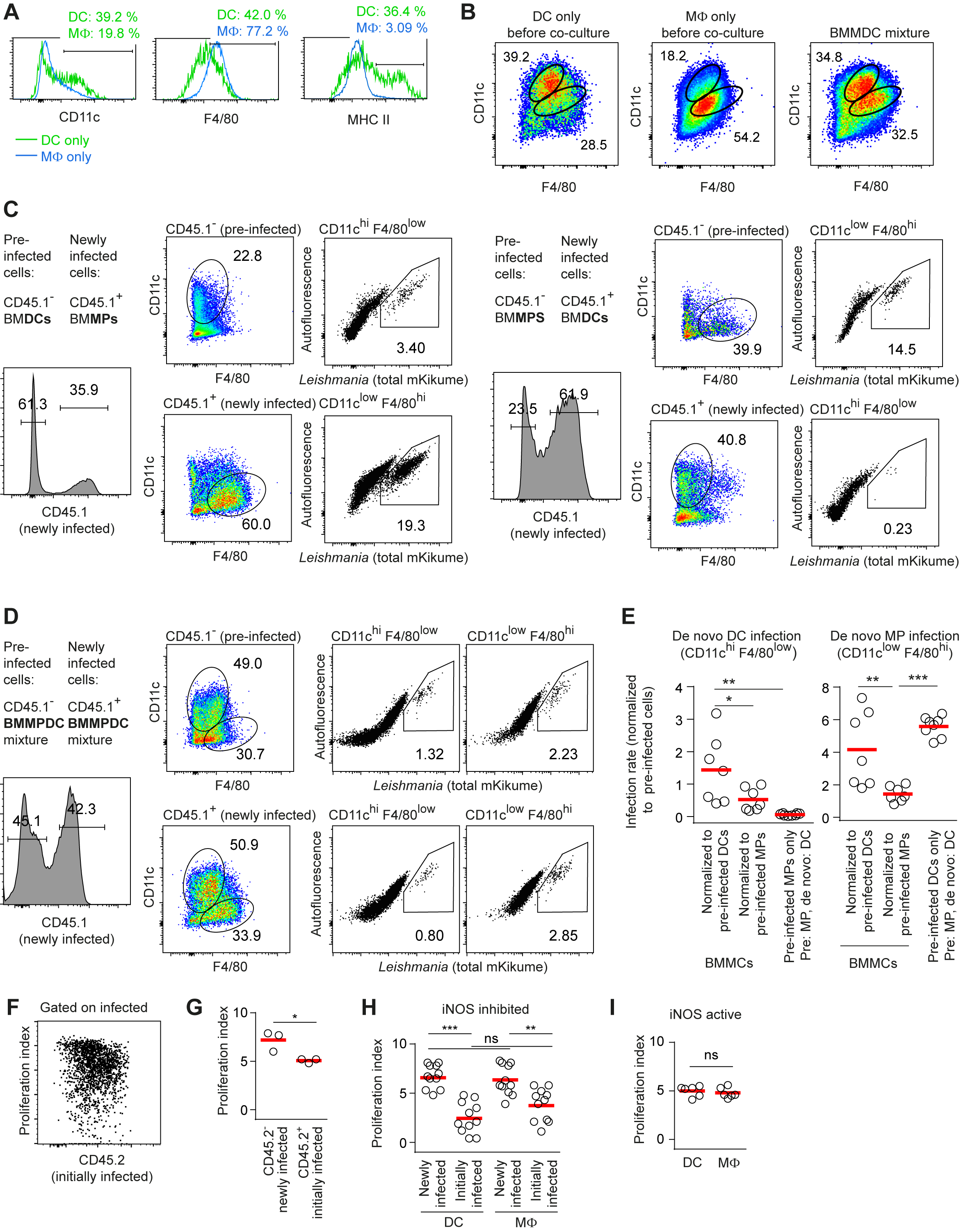

Supplement: S4 Fig — (A) Analysis of expression levels of uninfected CD11c, F4/80 and MHC class II in bone marrow-derived dendritic cell (green histograms) and macrophage (blue histograms) cultured. Data are representative of two independent experiments. (B) Analysis of CD11c versus F4/80 expression in infected dendritic cell (DC, left panel) and macrophage (MΦ, middle panel) and DC and MΦ mixed cultures (right panel). (C) Analysis of cell-to cell transfer efficiency from preinfected CD45.1- bone marrow-derived dendritic cells (BMDCs) into CD45.1+ bone marrow-derived macrophages (BMMPs) (left part) or CD45.1- BMMPs into CD45.1+ BMDCs (right part). (D) Analysis of cell-to cell transfer efficiency between mixed cultures of BMDCs and BMMPs (BMMPDC). (E) Quantitative comparison of infection rates in BMMCs (left graph) and BMDCs (right graph) as solely preinfected or newly recruited cells and in BMMPDC mixtures. Each symbol shows one individual experimental replicate. Data are pooled from two independent experiments. (F) Colour-switch control for L. major proliferation analysis in newly recruited cells, data shown are representative of three independent replicates (G) Quantification of L. major proliferation rates in newly infected (CD45.2-) and initially infected (CD45.2+) cells. Each symbol shows one individual experimental replicate. (H) Analysis of parasite proliferation in newly infected and initially infected cells under inhibition of the nitric oxide synthase iNOS by L-NIL and (I) in initially infected cells without inhibition of iNOS. ***p < 0.001; **p < 0.01; *p < 0.05; ns, not significant. Each symbol shows one individual experimental replicate. (TIF) [file ppat.1007374.s004.tif]

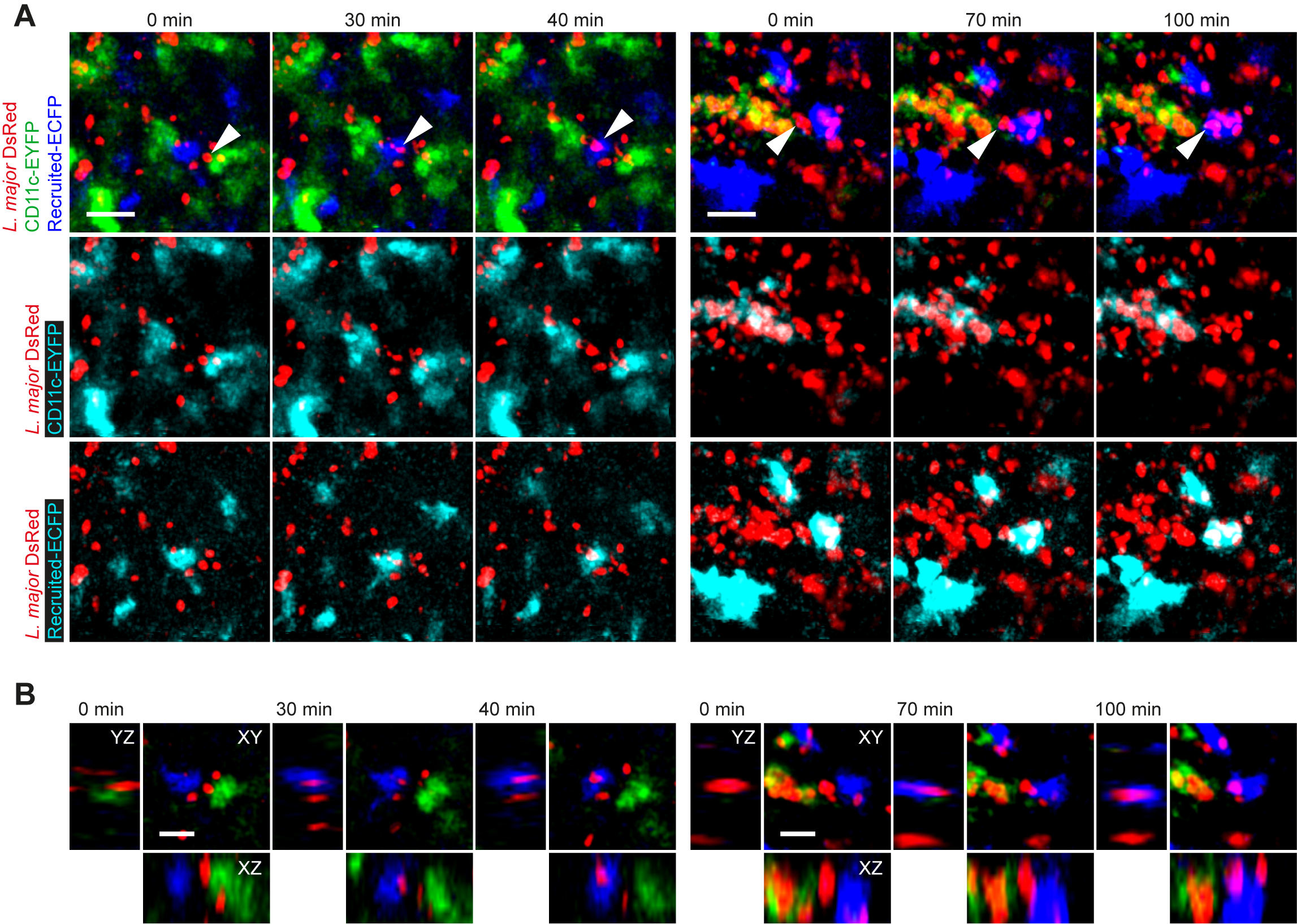

Supplement: S5 Fig — (A) Two examples de novo infection experiments of newly recruited cells (blue) by L. major (red) initially juxtapositioned to a CD11c+ host cell (green). Images are selected projections of 10–13 slices of 3 μm-spaced z-stacks taken longitudinally every 10 minutes. Individual color overlays of DsRed (red) with host CD11c-EYFP and the ECFP expressed by newly recruited cells are shown separately in the middle and bottom line of the panel. Scale bar, 20 μm. (B) XYZ-sections showing single imaging planes (XY) or reconstructions (XZ, YZ) of the image stacks shown in (B). Scale bar, 10 μm. (TIF) [file ppat.1007374.s005.tif]

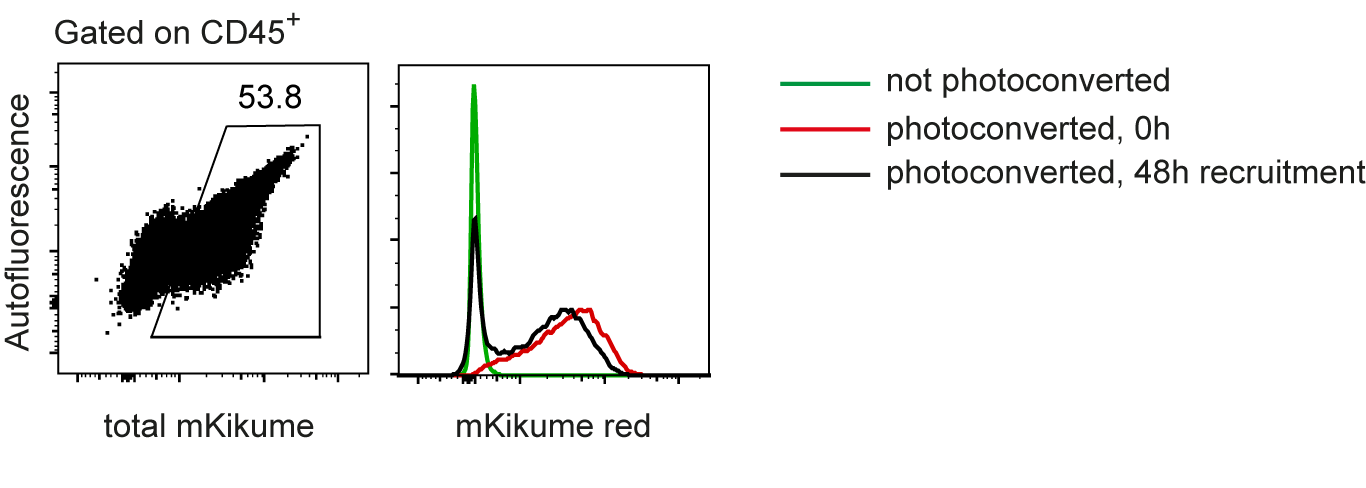

Supplement: S6 Fig — Ubiquitous mKikume expressing mice were infected with non-fluorescent L. major wild type. Photoconversion in the mouse ear was performed 48h prior to analysis. Control samples were photoconverted 0 h prior to analysis or not photoconverted at all. After gating on CD45+ cells, mKikume+ cells were identified. Cells which were photoconverted at the infection site 48h prior to analysis showed only a slight shift towards less red mKikume fluorescence, whereas non-photoconverted cells are recruited within this time period, indicating that metabolism-related recovery from photoconversion in mouse cells is not sufficient interfere with the identification of non-photoconverted, newly recruited cells. (TIF) [file ppat.1007374.s006.tif]
