# Supplementary material for: CD11c-expressing Ly6C+CCR2+ monocytes constitute a reservoir for efficient Leishmania proliferation and cell-to-cell transmission
Source: PLoS Pathog. 2018 Oct 22;14(10):e1007374. doi: 10.1371/journal.ppat.1007374 (PMC6211768; doi:10.1371/journal.ppat.1007374)
Supplement: S1 Table — Deconvolved 400 x 400 x 8 micron stacks were segmented with the RACE settings indicated. Three infection sites from different mice (Site1-Site3) and two Z planes per site (ZPl1-ZPl2) were converted into flow cytometry datasets and analyzed as described in the supplementary methods (see S1 Text). The number of total and infected cells detected at each site/plane is indicated in the upper part of the table, the rank within one plane and site is shown in the lower part. The optimized condition is boxed. (DOCX) [file ppat.1007374.s007.docx]

| **Absolute values** | |  |  |  |  |  |  |  |  |  |  |  |  |  |  |
| --- | --- | --- | --- | --- | --- | --- | --- | --- | --- | --- | --- | --- | --- | --- | --- |
| Max 2D Segment Area | Max. 3D Cell Volume | H-Maxima Level | Slice-by-Slice Watershed level | Total cells detected | | | | | | Infected cells detected | | | | | |
|  |  |  |  | Site1 | Site1 | Site2 | Site2 | Site3 | Site3 | Site1 | Site1 | Site2 | Site2 | Site3 | Site3 |
|  |  |  |  | ZPl1 | ZPl2 | ZPl1 | ZPl2 | ZPl1 | ZPl2 | ZPl1 | ZPl2 | ZPl1 | ZPl2 | ZPl1 | ZPl2 |
| 2000 | 8000 | 0.25 | 1 | 1389 | 1123 | 1559 | 1421 | 1460 | 1647 | 252 | 214 | 299 | 255 | 259 | 303 |
| 2000 | 8000 | 0.5 | 3 | 1595 | 1162 | 1642 | 1481 | 1524 | 1740 | 271 | 221 | 308 | 256 | 290 | 333 |
| 2000 | 8000 | 1 | 5 | 1138 | 777 | 1390 | 1130 | 1105 | 1460 | 241 | 127 | 248 | 239 | 186 | 257 |
| 3000 | 10000 | 0.25 | 1 | 1505 | 1165 | 1634 | 1804 | 1491 | 1716 | 267 | 220 | 325 | 304 | 281 | 326 |
| 3000 | 10000 | 0.5 | 3 | 1573 | 1190 | 1636 | 1500 | 1566 | 1760 | 280 | 224 | 318 | 271 | 300 | 331 |
| 3000 | 10000 | 1 | 5 | 1140 | 798 | 1468 | 1168 | 1148 | 1546 | 240 | 133 | 271 | 242 | 193 | 277 |
| 4000 | 12000 | 0.25 | 1 | 1529 | 1172 | 1653 | 1459 | 1519 | 1760 | 280 | 219 | 325 | 258 | 288 | 336 |
| 4000 | 12000 | 0.5 | 3 | 1549 | 1189 | 1711 | 1531 | 1541 | 1783 | 277 | 219 | 329 | 257 | 300 | 335 |
| 4000 | 12000 | 1 | 5 | 1170 | 802 | 1417 | 1180 | 1155 | 1506 | 250 | 131 | 258 | 247 | 191 | 267 |
|  |  |  |  |  |  |  |  |  |  |  |  |  |  |  |  |
| **Ranked values** | |  |  |  |  |  |  |  |  |  |  |  |  |  |  |
| Max 2D Segment Area | Max. 3D Cell Volume | H-Maxima Level | Slice-by-Slice Watershed level | Total cells detected | | | | | | Infected cells detected | | | | | |
|  |  |  |  | Site1 | Site1 | Site2 | Site2 | Site3 | Site3 | Site1 | Site1 | Site2 | Site2 | Site3 | Site3 |
|  |  |  |  | ZPl1 | ZPl2 | ZPl1 | ZPl2 | ZPl1 | ZPl2 | ZPl1 | ZPl2 | ZPl1 | ZPl2 | ZPl1 | ZPl2 |
| 2000 | 8000 | 0.25 | 1 | 6 | 6 | 6 | 6 | 6 | 6 | 6 | 6 | 6 | 6 | 6 | 6 |
| 2000 | 8000 | 0.5 | 3 | 1 | 5 | 3 | 4 | 3 | 4 | 4 | 2 | 5 | 5 | 3 | 3 |
| 2000 | 8000 | 1 | 5 | 9 | 9 | 9 | 9 | 9 | 9 | 8 | 9 | 9 | 9 | 9 | 9 |
| 3000 | 10000 | 0.25 | 1 | 5 | 4 | 5 | 1 | 5 | 5 | 5 | 3 | 2 | 1 | 5 | 5 |
| 3000 | 10000 | 0.5 | 3 | 2 | 1 | 4 | 3 | 1 | 2 | 1 | 1 | 4 | 2 | 1 | 4 |
| 3000 | 10000 | 1 | 5 | 8 | 8 | 7 | 8 | 8 | 7 | 9 | 7 | 7 | 8 | 7 | 7 |
| 4000 | 12000 | 0.25 | 1 | 4 | 3 | 2 | 5 | 4 | 2 | 1 | 4 | 2 | 3 | 4 | 1 |
| 4000 | 12000 | 0.5 | 3 | 3 | 2 | 1 | 2 | 2 | 1 | 3 | 4 | 1 | 4 | 1 | 2 |
| 4000 | 12000 | 1 | 5 | 7 | 7 | 8 | 7 | 7 | 8 | 7 | 8 | 8 | 7 | 8 | 8 |
|  |  |  |  |  |  |  |  |  |  |  |  |  |  |  |  |
|  |  | Condition rank: best | |  |  |  |  |  |  |  |  |  | worst | | |
