# Supplementary material for: CD11c-expressing Ly6C+CCR2+ monocytes constitute a reservoir for efficient Leishmania proliferation and cell-to-cell transmission
Source: PLoS Pathog. 2018 Oct 22;14(10):e1007374. doi: 10.1371/journal.ppat.1007374 (PMC6211768; doi:10.1371/journal.ppat.1007374)
Supplement: S2 Table — (DOCX) [file ppat.1007374.s008.docx]

| Antibody | Clone | Supplier | Concentration | Label |
| --- | --- | --- | --- | --- |
|  |  |  |  |  |
| ICAM-1 | YN1/1.7.4 | Biolegend | 1:120 | Alexa 488 |
| CD11b | M1/70 | eBioscience | 1:80 | Alexa 488 |
| CD45 | 30-F11 | BD Bioscience | 1:240 | FITC |
| F4/80 | BM8 | eBioscience | 1:240 | Alexa 488 |
| CD11c | N418 | eBioscience | 1:40 | Alexa 488 |
| CD45R | RA3-6B3 | BD Bioscience | 1:240 | Alexa 488 |
| CD11c | N418 | Biolegend | 1:80 | Alexa 488 |
| CD86 | GL-1 | Biolegend | 1:80 | Alexa 488 |
| MHCII | 2G9 | BD Bioscience | 1:500 | FITC |
| Propidium Iodide |  | Sigma | 1:5000 |  |
