# Supplementary material for: CD11c-expressing Ly6C+CCR2+ monocytes constitute a reservoir for efficient Leishmania proliferation and cell-to-cell transmission
Source: PLoS Pathog. 2018 Oct 22;14(10):e1007374. doi: 10.1371/journal.ppat.1007374 (PMC6211768; doi:10.1371/journal.ppat.1007374)
Supplement: S1 Text — (DOCX) [file ppat.1007374.s009.docx]

**Supplementary methods**

MELC image segmentation and analysis

Deconvolved and aligned image stacks (Z vs. XY ratio of 2.5 or 5) of CD11b, CD45 and CD54 stainings were used separately as membrane input, the corresponding propidium iodide staining served as seed input for the Real-time Accurate Cell-shape Extractor (RACE) program with ITK RACE acceleration, SSH Fusion Heuristic and the following settings: Max Threads: 16, Min Closing Radius: 1, Max Closing Radius: 4, Min Seed Area: 1, Min 3D Cell Volume: 3000, and Binary Threshold: 0.00080. Max 2D Segment Areas, Max 3D Cell Volumes, H-Maxima Levels and Slice-by-Slice Watershed Levels were varied according to Supplementary table 1 in order to identify a maximal number of infected cells, and found to be optimal at Max 2D Segment Area: 3000, Max 3D Cell Volume: 10000, H-Maxima Level: 0.5, and Slice-by-Slice Watershed Level: 3.

From the resulting image stacks of gray level-coded 2D objects, regions of interest (ROIs) were generated in three planes spaced 3 micron apart around the Z centre of the stacks using the Fiji software (NIH, https://imagej.nih.gov/ij/). CD11b-seeded stacks were processed first, subsequently, the ROIs were supplemented with RACE data from the CD45-seeded stacks, and finally from the CD54-seeded stacks. The cellular ROIs were used for mKikume fluorescence measurement. 2 pixel (0.4 micron) rim masks generated from the ROIs were used for surface marker measurements. The mean fluorescence of the cellular and rim ROIs were extracted for each cell and fluorescence channel. In order to normalize image-to-image differences in background and overall staining intensity, the individual ROI fluorescence values were normalized

$100+\left( 100* \frac{{Fluorescence}_{ROI} - {Fluorescence}_{20th Percentile, all ROIs in image}}{{Fluorescence}_{80th Percentile, all ROIs in image}} \right)_{rounded to the nearest integer}$

with the factor and constant 100 introduced in order to obtain positive integers as fluorescence values. The resulting data tables were converted into .fcs files and analysed using the FlowJo software (FlowJo LCC).

For defining fluorescence thresholds for gating within FlowJo, 30 marker-positive and 30 marker-negative cells were selected from images of three different sites of infection and a high-specificity cutoff was defined (i.e. no marker-negative cells in the positive gate).
